# PCDH1 promotes progression of pancreatic ductal adenocarcinoma via activation of NF-κB signalling by interacting with KPNB1

**DOI:** 10.1038/s41419-022-05087-y

**Published:** 2022-07-21

**Authors:** Zhihua Ye, Yingyu Yang, Ying Wei, Lamei Li, Xinyi Wang, Junkai Zhang

**Affiliations:** Department of Medical Oncology Center, Zhongshan City People’s Hospital, 528403 Zhongshan City, Guangdong Province P. R. China

**Keywords:** Oncogenes, Prognostic markers

## Abstract

Uncontrolled growth, distant metastasis and chemoresistance are critical characteristics of pancreatic ductal adenocarcinoma (PDAC), and they result in high mortality; however, the mechanisms triggering these effects have not been fully investigated. In this study, we analysed a dataset in the Cancer Genome Atlas (TCGA) and identified PCDH1, a rarely studied transmembrane protein, as a novel prognostic marker in PDAC patients. We demonstrated that PCDH1 expression was upregulated in PDAC tissues, and its expression levels were associated with the depth of tumour invasion and lymph node metastasis. Patients with high PCDH1 levels showed poor overall survival (OS). We also investigated the biological significance of PCDH1 in PDAC cell growth, metastasis, and side population (SP) phenotype acquisition and explored the internal molecular mechanisms of PCDH1 action. Our results demonstrated that PCDH1 enhanced p65 nuclear localization by interacting with KPNB1, a well-characterized nuclear transporter, thereby activating the NF-κB signalling pathway and increasing its functional effects during PDAC progression. Hence, our results indicate that PCDH1 can be used as a negative prognostic marker and may be a potential therapeutic target for PDAC patients.

## Introduction

Pancreatic ductal adenocarcinoma (PDAC) is one of the most lethal malignancies, accounting for more than 90% of pancreatic cancer patients worldwide, with a 5-year survival rate lower than 10% [1, 2]. Due to the lack of specific symptoms and absence of efficient methods for early detection, more than 80% of PDAC patients present with have regional or distant metastasis upon initial diagnosis [3]. Moreover, PDAC has been shown to be highly refractory to conventional chemotherapy or combined treatment with other newly developed agents [4, 5]. Therefore, it is of great importance to find diagnostic and therapeutic targets and to elaborate the molecular mechanisms of PDAC carcinogenesis.

Increasing evidence suggests that persistent activation of the NF-κB signalling pathway plays a critical role in the tumorigenesis of pancreatic cancer and confers chemoresistance to tumour cells [6, 7]. In addition, inhibition of NF-κB signalling increases the apoptosis rate of PDAC cells [8]. In contrast, blocking constitutive NF-κB activity in the Panc-1 PDAC cell line by overexpressing phosphorylation-defective IκBα dramatically downregulated the expression of IL-8 and VEGF, resulting in the suppression of PDAC progression [9]. Hence, further studies on the regulatory mechanisms of the NF-κB signalling pathway in pancreatic cancer are of great significance.

Protocadherins are transmembrane proteins in the cadherin superfamily and are subgrouped into clustered and nonclustered protocadherin categories [10–12]. Aberrant expression and genetic alteration of certain family members are associated with neurological disorders and cancer in humans. Previous studies have proven that PCDH1 is indispensable for the entry of new world hantavirus into cells [13] and plays a role in maintaining airway epithelial integrity [14].

Herein, we report that a high level of PCDH1 in PDAC is closely correlated with poor prognosis. Both in vitro and in vivo experiments demonstrated that PCDH1 upregulated expression promotes the proliferation and metastasis of PDAC cell lines. Further molecular studies revealed that PCDH1 interacts with KPNB1, which enhances p65 nuclear import and activated the NF-κB pathway in PDAC cells. Thus, our findings suggest that PCDH1 is a potential target in PDAC prognostics and therapeutics.

## Results

### PCDH1 expression is upregulated in PDAC and predicts poor survival

**Fig. 1:**
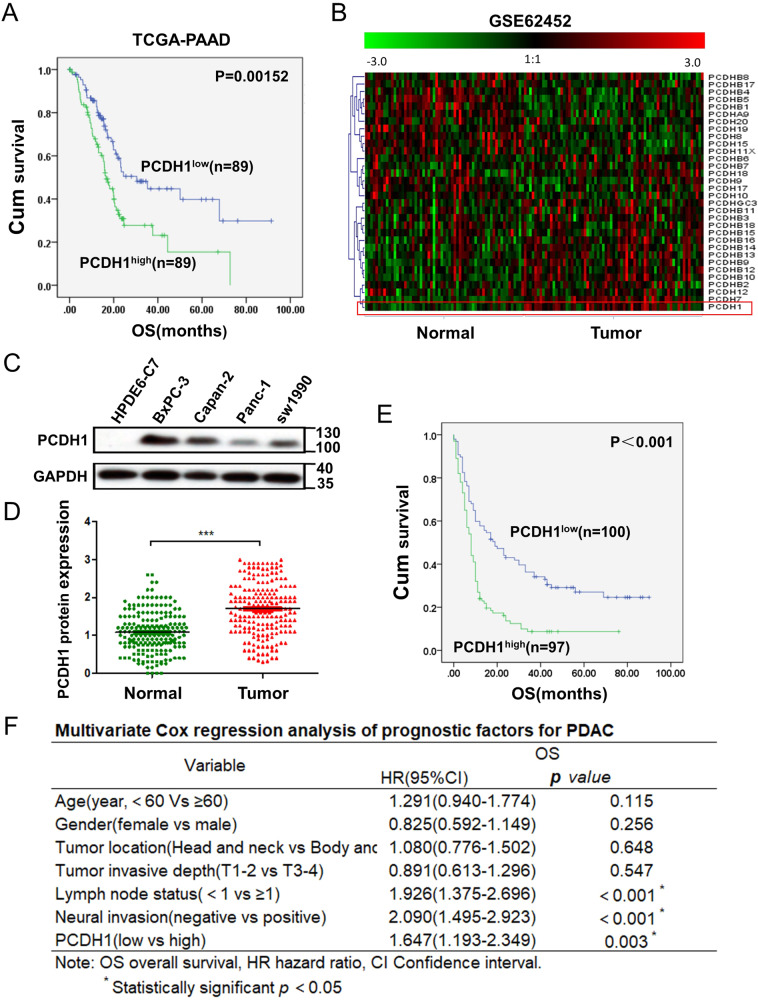
PCDH1 expression is upregulated in PDAC and predicts poor survival. **A** High expression of *PCDH1* in the TCGA dataset was correlated with worse prognosis in PDAC. **B** Heatmap of 32 genes of the protocadherin family in PDAC and noncancerous tissues. **C** In the HPDE6-C7 normal pancreatic ductal epithelial cell line and four PDAC cell lines, the expression of PCDH1 was detected by Western blotting. **D** Immunohistochemical (IHC) analysis of PCDH1 expression in 197 PDAC tissues and adjacent normal pancreatic tissues. The data were assessed by paired-samples *t* test. **E** Kaplan–Meier survival analysis of the correlation between PCDH1 expression and OS (log-rank test). **F** Multivariate Cox regression analysis of the prognostic factors of PDAC patients. ***P < 0.001.

To identify new tumour markers for pancreatic cancer, we analysed the online RNA sequencing data combined with survival data obtained with 178 PDAC specimens from The Cancer Genome Atlas (TCGA) database using the GEPIA online program [15]. We found that the expression of *PCDH1* in PDAC was negatively correlated with patient overall survival (OS) (Fig. 1A). Moreover, we assessed microarray data from the Gene Expression Omnibus (GEO) GSE62452 dataset [16] to compare the expression of *PCDH1* in tumour and normal tissues. We found that the abundance of *PCDH1* mRNA was higher in the GEO tumour tissues. Additionally, *PCDH1* was the most significantly upregulated gene among protocadherins (Fig. 1B). Next, we examined PCDH1 expression at the protein level. Western blotting revealed that the expression of PCDH1 in PDAC cell lines was much higher than that in the HPDE6-C7 normal pancreatic duct epithelial cell line (Fig. 1C). Immunohistochemistry (IHC) staining of PCDH1 in 197 histopathologically confirmed PDAC tissues was performed, and the histochemistry score (H score) [17] was determined for each tumour tissue and adjacent normal pancreatic tissue (4 degrees, Fig. S1). The results showed that PCDH1 was localized to the cytoplasm and cytomembrane and that its expression was significantly higher in the tumour tissues than in the normal tissues (Fig. 1D).

High PCDH1 expression was detected in 97 (49.2%) PDAC samples, and low PCDH1 expression was observed in 100 (50.8%) samples. To understand the clinical relevance of PCDH1 expression in PDAC, the relationship between PCDH1 levels and the clinicopathological variables of PDAC patients was analysed. The results showed that PCDH1 expression was significantly associated with the depth of tumour invasion (*P* = 0.023) and lymph node metastasis (*P* = 0.015) (Table 1). A high level of PCDH1 expression indicated the presence of extensive tumour cell infiltration and lymph node metastasis. A Kaplan–Meier survival analysis was performed to assess the association between PCDH1 levels and patient survival data. The results showed that the expression of PCDH1 was negatively correlated with patient OS (*P* < 0.001, Fig. 1E), consistent with the TCGA data (Fig. 1A). Furthermore, we performed a multivariate Cox regression analysis on the clinical parameters of the patients. The HR values indicating lymph node metastasis, degree of neural invasion and PCDH1 expression with respect to the OS of the patients were 1.926, 2.09 and 1.647, respectively (*P* < 0.05) (Fig. 1F), indicating that PCDH1 expression is an independent prognostic factor for the OS of PDAC patients.

**Table 1:** Clinicopathological findings and correlation with PCDH1 expression.

Varialbes	No.(%)	PCDH7 low	PCDH7 high	*P* value
Total cases	197	100(50.8)	97(49.2)	
*Age(years)*				
<60	100(50.8)	50(25.4)	50(25.4)	
≥60	97(49.2)	47(25.9)	50(25.4)	0.828
*Gender*				
Female	88(44.7)	45(22.8)	43(21.8)	
Male	109(55.3)	52(26.4)	57(28.9)	0.632
*Tumour location*				
Head and neck	128(65.0)	60(30.5)	68(34.5)	
Body and tail	69(35.0)	37(18.8)	32(16.2)	0.366
*Tumour invasive depth*^*a*^				
T1–2	133(67.5)	58(29.4)	75(38.1)	
T3–4	64(32.5)	39(19.8)	25(12.7)	0.023^b^
*Lymph node status*				
<1	117(59.4)	66(33.5)	51(25.9)	
≥1	80(40.6)	31(15.7)	49(24.9)	0.015^b^
*Neural invasion*				
Negative	112(57.9)	60(30.5)	52(26.4)	
Positive	85(42.1)	37(18.8)	48(24.4)	0.163

^a^According to the 8th Edition of the AJCC Cancer Staging Manual.

^b^Statistically significant, *P* < 0.05.

### PCDH1 promotes the growth, metastasis and side population phenotype of PDAC cells in vitro

**Fig. 2:**
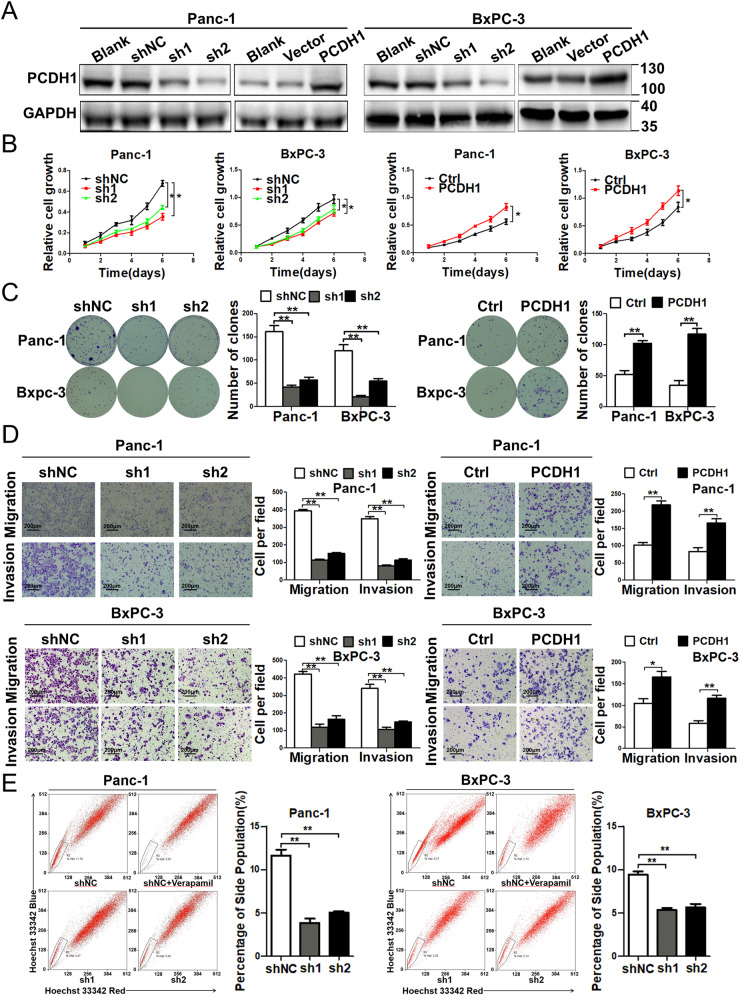
PCDH1 promotes the growth, metastasis and side population phenotype acquisition of PDAC cells in vitro. **A** Knockdown efficiency and enforced expression of PCDH1 in PDAC cell lines was evaluated by Western blotting. **B** An MTT assay was performed to detect the effect of PCDH1 on the proliferation of PDAC cell lines. **C** Representative images showing the effects of PCDH1 expression knockdown (left) or overexpression (right) on colony formation. The number of colonies containing more than 50 cells was counted. **D** The effects of PCDH1 knockdown (left) or overexpression (right) on migration and invasion were evaluated by Transwell assays. **E** Representative images showing the effects of PCDH1 knockdown on SP phenotype acquisition. The results are presented as the mean ± SD of three independent experiments. The data were assessed by Student’s 2-tailed *t* test. *P < 0.05, **P < 0.01.

To specify the relevant mechanism through which PCDH1 affects the progression of PDAC, PCDH1 expression was knocked down by short hairpin RNA (shRNA) or PCDH1 was ectopically expressed in Panc-1 and BxPC-3 cells (Fig. 2A). An MTT assay showed that silencing PCDH1 remarkably attenuated the growth of the Panc-1 and BxPC-3 cells, while enforced PCDH1 expression enhanced the proliferation of both cell lines (Fig. 2B). Additionally, a subsequent colony formation assay confirmed these results (Fig. 2C). Transwell assays revealed that PCDH1 overexpression or knockdown greatly enhanced or impaired the migration and invasion of the Panc-1 and BxPC-3 cells (Fig. 2D). We further investigated the role played by PCDH1 in regulating the side population (SP) phenotype, which has been identified in cultured pancreatic cancer cells and shows similar characteristics to cancer stem cells (CSCs) [18, 19]. First, we performed a flow cytometry analysis to measure the proportion of cells with the SP phenotype among PCDH1-silenced Panc-1 and BxPC-3 cells. The results showed that downregulating PCDH1 expression significantly suppressed the acquisition of the SP phenotype in both cell lines (Fig. 2E). In contrast, the upregulated expression of PCDH1 greatly enhanced the number of cells Panc-1 and BxPC-3 cells acquiring the SP phenotype (Fig. S2A). Next, the SP cells and non-SP cells among PDAC cells were sorted, and endogenous expression of PCDH1 was detected. Compared to that in the non-SP cells, PCDH1 expression was upregulated in the SP cells (Fig. S2B). Therefore, our results provide evidence showing that high PCDH1 expression in PDAC promotes acquisition of the SP phenotype, an inherent stem cell characteristic correlated with tumour growth, invasion and metastasis.

### PCDH1 promotes the growth and metastasis of PDAC cells in vivo

**Fig. 3:**
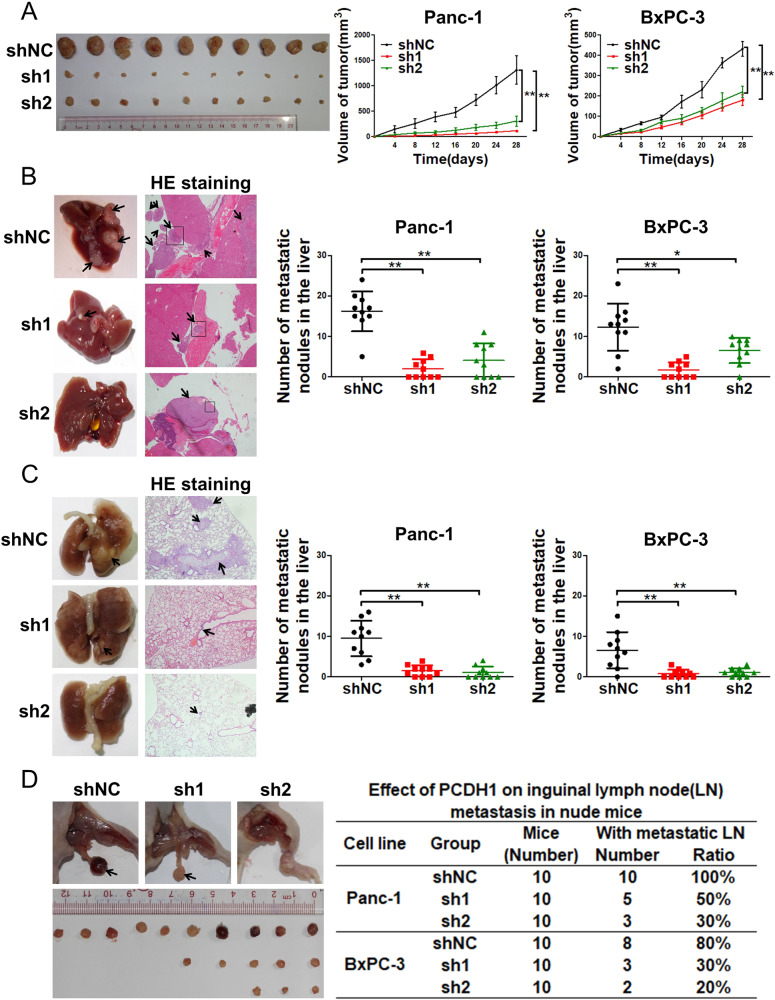
PCDH1 promotes the growth and metastasis of PDAC cells in vivo. PCDH1-downregulated PDAC cells were injected into nude mice on the basis of the different model established ((**A**) refers to the xenograft model; (**B**) refers to the hepatic metastasis model; (**C**) refers to the lung metastasis model; and (**D**) refers to the lymph node metastasis model). Each group contained 10 nude mice. **A** The growth of the subcutaneous xenograft tumours derived from the PDAC cells implanted in the nude mice (left, representative images showing the xenografts removed on day 28; right, volume of the tumours at different times). **B** Representative microscopy images of livers and haemoxylin and eosin (HE)-stained metastatic nodules (left). Number of metastases per liver (right). **C** Representative microscopy images of lungs and HE-stained metastatic nodules (left). Number of metastases per lung (right). **D** Lymph node metastases in the nude mice after intraplantar injection (left, representative images; right, the number and ratio of mice with lymph node metastasis). The results are presented as the mean ± SD of three independent experiments. The data were assessed by Student’s 2-tailed *t* test. *P < 0.05, **P < 0.01.

To explore the role played by PCDH1 in vivo, we first established a xenograft model using PCDH1-silenced Panc-1 and BxPC-3 cells subcutaneously injected into the flanks of BALB/c nude mice. The results showed that the volume of the tumours generated from PCDH1-silenced Panc-1 and BxPC-3 cells was dramatically smaller than that of the control groups (Fig. 3A). Next, we established a hepatic metastasis model by injecting PCDH1-silenced PDAC cells into the spleens of nude mice, as reported previously [20]. Silencing PCDH1 expression resulted in a significant reduction in the number of liver metastases (Fig. 3B), and a similar effect was observed with a lung metastasis model (Fig. 3C). As we previously mentioned, PCDH1 expression correlates with lymph node metastasis in PDAC tissues, and we further generated a lymph node metastasis model with nude mice. The results showed fewer metastatic inguinal lymph nodes in the mice after PCDH1 downregulation (Fig. 3D). Taken together, these results suggest that PCDH1 facilitates the proliferation and metastasis of PDAC cells in vivo.

### PCDH1 functions by activating the NF-κB pathway in PDAC

**Fig. 4:**
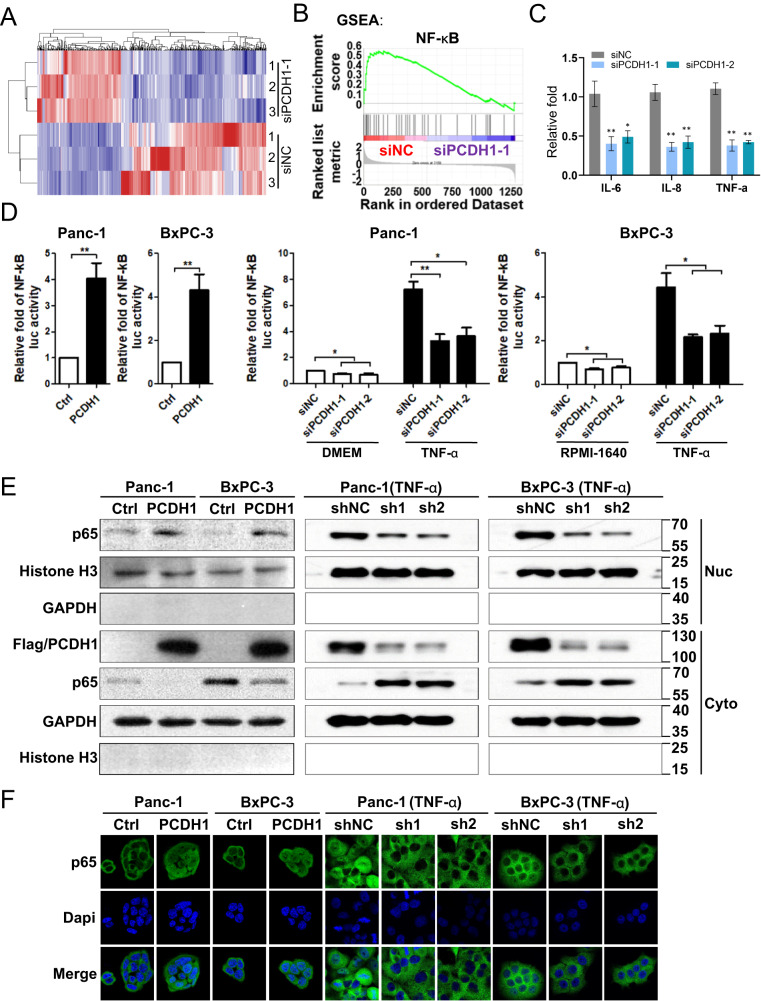
PCDH1 activates the NF-κB pathway in PDAC cells. **A** Representative heatmaps showing the differentially expressed genes (fold change > 2) after PCDH1 silencing by RNA interference in Panc-1 cells and the control group. The expression levels are shown as log2-transformed values. **B** GSEA of mRNA profiles revealed inhibited expression of NF-κB target genes after PCDH1 silencing by RNA interference in Panc-1 cells compared with the control group. **C** Quantitative RT–PCR showing that NF-κB regulated IL-6, IL-8 and TNFα mRNA expression in Panc-1 cells with in PCDH1 silenced by RNA interference and the control group. **D** Dual-luciferase assays were performed to detect the effects of PCDH1 on the NF-κB signalling pathways in PDAC cell lines. Western blot assay (**E**) and immunofluorescence assays (**F**) were performed to detect the effect of PCDH1 on the nuclear localization of p65. For the PCDH1 silencing groups shown in Figs. 4D, 4E and 4F, TNF-α (20 ng/ml) (Sigma–Aldrich, GF314) pretreatment was performed for 10 min. The results are presented as the mean ± SD of three independent experiments. The data were assessed by Student’s 2-tailed *t* test. *P < 0.05, **P < 0.01.

To explore the molecular mechanisms by which PCDH1 contributes to PDAC progression, we used a microarray to detect gene expression profiling of PCDH1 silencing in Panc-1 cells induced by RNA interference (Fig. 4A). To search for specific signalling pathways, a gene set enrichment analysis (GSEA) of the transcriptome was conducted. Figure 4B shows that NF-κB expression was inhibited in PCDH1-silenced cells, confirming the significant role played by PCDH1 in NF-κB pathway activation. This finding was validated by qRT–PCR assays showing that the expression of NF-κB-mediated cytokines, including IL-6, IL-8 and TNF-α, was downregulated in PCDH1-silenced cells (Fig. 4C). Moreover, dual-luciferase reporter assays showed that exogenous overexpression of PCDH1 significantly enhanced the activity of the NF-κB pathway (Fig. 4D, left), while silencing PCDH1 expression inhibited the NF-κB pathway with or without TNF-α activation (Fig. 4D, right). However, no effects on the SRE, STAT3, AP-1, TOP-1, p53 or Rb reporter were observed (data not shown). Together, these data confirm the significant role played by PCDH1 in NF-κB pathway activation.

Next, we detected the expression of p65, a recognized NF-κB transcription factor, in both the cytoplasm and nucleus of PDAC cells. The overexpression of PCDH1 significantly promoted the nuclear localization of p65, while PCDH1 silencing inhibited this process (Fig. 4E). We also performed p65 immunofluorescence staining with Panc-1 and BxPC-3 cells overexpressing PCDH1 or a control vector. Our results showed that in the absence of extracellular stimulation (serum-free medium), p65 resided mainly in the cytoplasm in the control group. However, in the PCDH1-overexpressing group, higher levels of p65 were observed in the nucleus. In addition, after TNF-α pretreatment, part of p65 entered the nucleus, and silencing PCDH1 could inhibit this process (Fig. 4F). Considering that phosphorylation of IKK and IκBα are key regulatory steps in NF-κB pathway activation, we detected the phosphorylation levels IKK and IκBα by western blotting. Interestingly, we found that silencing of PCDH1 expression did not affect the phosphorylation levels of IKK or IκBα (Fig. S3), suggesting that PCDH1 regulates p65 at a position further downstream of IKK and IκBα in the NF-κB pathway.

**Fig. 5:**
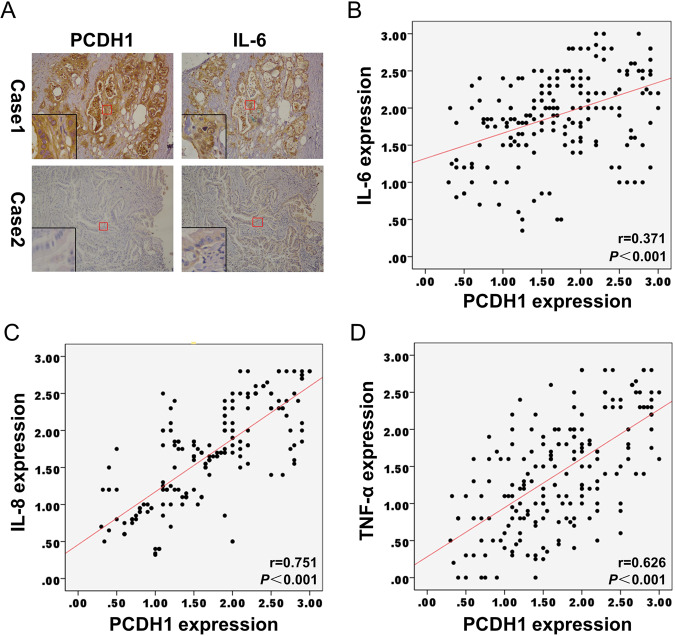
Correlation between the expression of PCDH1 and NF-κB downstream effectors in 197 PDAC tissues. **A** Representative IHC staining for PCDH1 and IL-6 is shown in two PCAC tissues. Correlations were detected between IL-6 (**B**), IL-8 (**C**), TNF-α (**D**) and PCDH1 expression by Pearson’s test in 197 PDAC tissues.

Since IL-6, which is a widely studied NF-κB effector, is closely related to tumour progression [21], we sought to determine whether its expression level was associated with PCDH1 in PDAC tissues. IHC staining for IL-6 in the 197 PDAC tissues described above was performed; representative images are shown in Fig. 5A. IL-6 expression was positively correlated with PCDH1 expression (Fig. 5B, *r* = 0.371, *P* < 0.001). Moreover, IHC staining of two other NF-κB effectors, IL-8 and TNF-α, showed similar results (Fig. 5C, *r* = 0.751, *P* < 0.001 and Fig. 5D, *r* = 0.626, *P* < 0.001). In addition, NF-κB downstream effectors, such as CCND1, CCNE2, MET, VEGFA, CD44 and CD133, contribute to cancer proliferation, metastasis and stemness [22–28], and their expression is regulated by PCDH1 (Fig. S4). Analysis of the TCGA dataset (via GEPIA) showed that *PCDH1* mRNA expression was positively correlated with the expression of the aforementioned genes in PDAC tissues (Fig. S5). In summary, PCDH1 activates the NF-κB pathway in PDAC cell lines and tissues.

To further verify NF-κB pathway mediate the transforming activity of PCDH1, we co-transfected PCDH1 plasmid and siRNA targeting p65 into PDAC cells, and then MTT assays and Transwell assays were conducted. Compared with the siNC groups, we found that the ability of PCDH1 to promote PDAC cells proliferation and migration was significantly attenuated after p65 silencing, indicating that PCDH1 exerts it biological effects through NF-κB pathway (Fig. S6A, B).

### PCDH1 activates the NF-κB pathway during the progression of PDAC by interacting with KPNB1

**Fig. 6:**
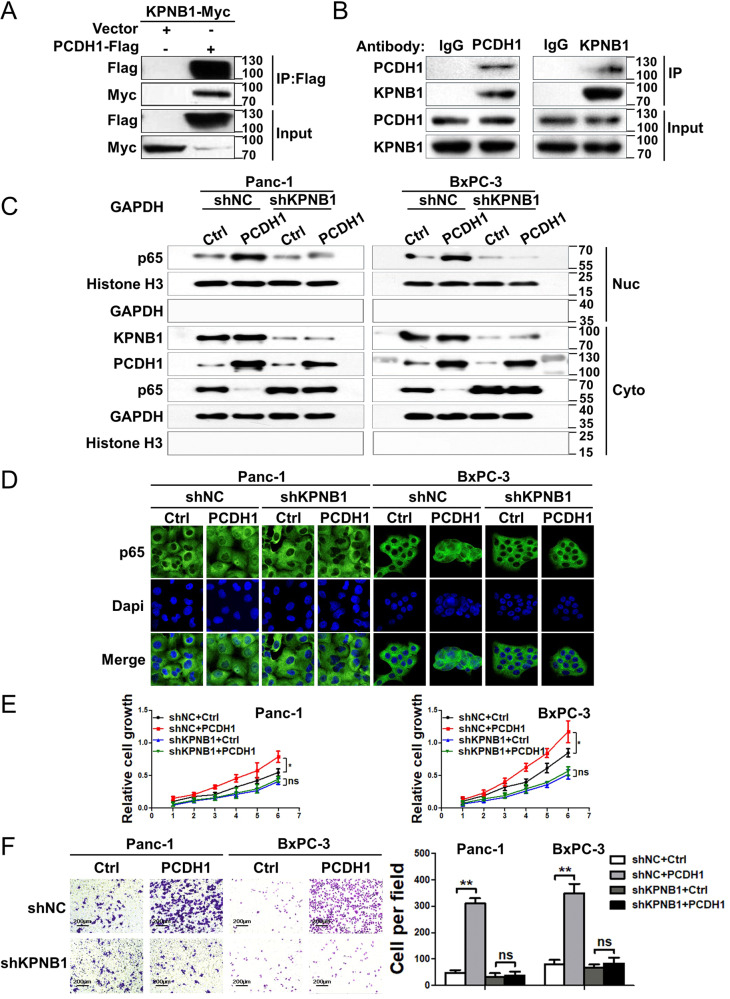
PCDH1 activates the NF-κB pathway during PDAC progression by interacting with KPNB1. **A** Coimmunoprecipitation (co-IP) and Western blot assays showing exogenous PCDH1 and KPNB1 expression in Panc-1 cells. **B** Co-IP and Western blot assays showing endogenous PCDH1 and KPNB1 expression in Panc-1 cells. Western blot assay (**C**) and immunofluorescence assays (D) were performed to detect the effect of PCDH1 on the nuclear localization of p65 after KPNB1 silencing. MTT (**D**) and Transwell assays (**E**) were performed to detect the effects of PCDH1 expression on PDAC cell proliferation and migration after KPNB1 silencing. The results presented are the mean ± SD of three independent experiments. The data were assessed by Student’s 2-tailed *t* test. *P < 0.05, **P < 0.01.

To elucidate the specific mechanism by which PCDH1 functions as an NF-κB activator, a mass spectrometry experiment was performed with Panc-1 cells overexpressing pcDNA3.1-PCDH1-Flag or a control vector to identify proteins that interact with PCDH1. Candidate proteins ranked according to the analysis scores are presented in Table S1. The results showed that PCDH1 scored highest on mass spectrometry, indicating that the coimmunoprecipitation (co-IP) experiment had been successful. Among these candidate proteins, we found that KPNB1, a major nuclear receptor involved in shuttling proteins from the cytoplasm to the nucleus, including p65, activated the NF-κB pathway [29]. For verification, an exogenous co-IP assay after cell cotransfection of plasmids expressing Flag-tagged PCDH1 and Myc-tagged KPNB1 was performed. The results showed that KPNB1 was pulled down by PCDH1 (Fig. 6A). A co-IP assay with Panc-1 cells showed that endogenous PCDH1 and KPNB1 interacted with each other (Fig. 6B).

To specify whether PCDH1 plays the biological role mentioned above by binding KPNB1, we first constructed KPNB1-silenced cell lines in Panc-1 and BxPC-3 cells. Next, we overexpressed PCDH1 or control vector in KPNB1-silenced cells and measured the level of nuclear localization of p65 by Western blot. Compared with the shNC groups, we found that the ability of PCDH1 to promote p65 nuclear import was significantly reduced in KPNB1-silenced PDAC cells (Fig. 6C). We also performed p65 immunofluorescence staining and found that the results were consistent with the aforementioned findings showing that PCDH1 activation of the NF-κB pathway in PDAC cells was dependent on its interaction with KPNB1 (Fig. 6D). Additionally, MTT assays and Transwell assays revealed that the ability of PCDH1 to promote PDAC cells proliferation and migration was significantly attenuated after KPNB1 expression was downregulated (Fig. 6E, F). Previous studies have shown that KPNB1, as an important nuclear transporter, can bind to dissociated p65 and help p65 transfer into the nucleus. Further experiments showed that the deletion of PCDH1 by specific siRNA reduced p65 binding to KPNB1 (Fig. S7). Collectively, these experimental findings established that PCDH1 functions as an oncoprotein in PDAC cells by interacting with KPNB1.

## Discussion

Protocadherins differ from classic cadherins in many respects [30]. Notably, protocadherin family members play diverse roles in various cancer types. For instance, PCDH8 and PCDH20 are considered tumour suppressors, while PCDH11Y functions as an oncoprotein [31–33]. Nevertheless, the function of PCDH1 in cancers has rarely been reported. By analysing the TCGA database, we found that *PCDH1* plays a possible role as an oncogene in PDAC. Further experiments demonstrated that PCDH1 was frequently expressed in PDAC cell lines and tissues, and its expression level was associated with the depth of tumour invasion and lymph node metastasis. PCDH1 may be an independent predictor for the survival of PDAC patients.

Sustained proliferation, activation of cell invasion and metastasis, and induction of angiogenesis are hallmarks of cancer [34]. CSCs are key chemotherapy resistance and may be the main cause of metastasis [35]. Our findings demonstrated that PCDH1 promotes PDAC cell proliferation and migration and the acquisition of the CSC phenotype in vitro. Additionally, experiments with nude mice showed that silencing PCDH1 expression significantly reduced blood and lymphatic metastasis of PDAC cells in vivo. By analysing the TCGA database, we found that the expression of PCDH1 was positively correlated with CCND1, CCNE2, VEGFA, MET, CD44 and CD133 expression in pancreatic cancer tissues. Overexpression and interference of PCDH1 expression significantly enhanced and inhibited, respectively, the expression of the aforementioned genes in PDAC cells. It has been widely reported that these genes are closely related to the proliferation, metastasis and stemness of malignant tumour cells. Therefore, PCDH1 plays an oncogenic role in PDAC development and can be considered a potential therapeutic target in the future.

Considering evidence has shown that NF-κB is constitutively activated in PDAC and other malignant cancers, inducing cell proliferation, invasion, angiogenesis, inflammation and stemness [36–42]. Aberrant activation of the NF-κB pathway also contributes to acquired resistance to gemcitabine [7, 43]. In addition, the abovementioned genes regulated by PCDH1 are associated with the malignant transformation of PDAC and are all downstream effectors of the NF-κB pathway. Our results demonstrated that PCDH1 promoted p65 nuclear import, which activated the NF-κB pathway in pancreatic cancer cells and tissues, and that this activation was achieved through PCDH1 interaction with KPNB1, a widely studied nuclear transporter. After silencing KPNB1 expression, the ability of PCDH1 to activate NF-κB signalling was abrogated, blocking the oncogenic effects of PCDH1.

Although our research has proved important results, some limitations need to be mentioned. Previous studies have suggested that KPNB1 plays an important role in the malignant transformation of head and neck, lung and gastric cancer. Inhibiting KPNB1 expression can significantly reduce the proliferative and migratory ability of tumour cells and induce apoptosis [44]. However, no evidence indicates that KPNB1 plays the same role in pancreatic cancer, and the relationship between KPNB1 and SP phenotype acquisition remains unknown. By analysing the TCGA database, we found that the prognosis of PDAC patients with high KPNB1 expression was worse (Fig. S8), suggesting that *KPNB1* functions as an oncogene in pancreatic cancer. Further studies need to be carried out to confirm this finding in the future. In addition, although we found that silencing of PCDH1 impaired the binding of p65 and KPNB1, which may be associated with the phenomenon that PCDH1 promotes p65 nuclear import, the more specific mechanism remains undefined. Furthermore, chemotherapy resistance is a main cause of poor prognosis in pancreatic cancer, and CSCs are closely related to this resistance. The study of PCDH1 and gemcitabine resistance is worth further study to fully assess the value of PCDH1 as a molecular marker for PDAC.

In conclusion, the dysregulation of PCDH1 expression plays a pivotal role in the progression of PDAC. We demonstrate that PCDH1 expression is upregulated in PDAC and promotes PDAC development by elucidating the potential mechanism of PCDH1 action. This study shows that PCDH1 is a promising marker and potential therapeutic target in PDAC.

## Materials and methods

### Bioinformatics analysis of gene expression in PDAC based on online datasets

The expression of *PCDH1* in PDAC tissue and control pancreatic tissues was determined with the GEO dataset GSE62452 [16]. Analysis of the TCGA dataset was performed using the GEPIA online program (http://gepia.cancer-pku.cn/) [15].

### Immunohistochemical (IHC) staining

Patient tissue specimens and selection criterion was described in the Supplementary Methods and Materials. IHC staining for PCDH1 (1:400, #HPA050538), IL-6 (1:200, #SAB4301665), TNF-α (1:100, #SAB4502982) (Sigma–Aldrich, St. Louis, MO, USA) and IL-8 (1:200, #94407) (CST, MA, USA) was performed as previously reported [45]. The expression of PCDH1, IL-6, IL-8 and TNF-α was independently evaluated by two pathologists. A receiver operating characteristic (ROC) curve analysis was performed to determine a cut-off value for PCDH1 high expression and low expression. An H score of 1.65 was thus used as the cut-off value.

### Cell lines, siRNAs, plasmids and transfections

The HPDE6-C7 normal pancreatic ductal epithelial cell line was a gift from Prof. Dongxin Lin [46]. The other cell lines were obtained from the American Type Culture Collection (ATCC) and were cultured as directed by the ATCC. Panc-1 and BxPC-3 were recently authenticated by STR profiling, and no mycoplasma contamination was detected. siRNAs (Table S2) were synthesized by RuiBiotech (Beijing, China). *PCDH1* and *KPNB1* full-length cDNA sequences with a Flag or Myc tag were inserted into pcDNA3.1. siRNAs and plasmids were transfected using Lipofectamine 3000 (Invitrogen, Carlsbad, CA, USA).

### Cell line construction

Full-length cDNA of *PCDH1* was cloned into a pCDH-EF1-MCS-T2A-Puro vector. shRNAs targeting *PCDH1* and *KPNB1* and a scramble shRNA were inserted into a plko.1 vector. These shRNAs were co-transfected with pspAX2 and pMD2G into 293T cells to generate lentivirus, and the infected cells were then harvested after 24 h. The lentivirus supernatant was used to infect Panc-1 and BxPC-3 cells, and puromycin (Sigma-Aldrich) was added 24 h after infection to select for stable cell lines. The sequences of the oligonucleotides targeting *PCDH1* and *KPNB1* are listed in Table S2.

### qRT–PCR

Total RNA was extracted from PDAC cells using TRIzol (Invitrogen), and cDNA was synthesized using M-MLV reverse transcriptase (Thermo Fisher) according to the manufacturer’s instructions. Real-time PCR was performed using a SYBR Green PCR kit (Sigma–Aldrich). The housekeeping gene *GAPDH* was used as the endogenous control. The 2-△△CT method was used to analyse the relative changes in gene expression. The primer sequences are presented in Table S3.

### Immunoprecipitation (IP) and Western blot analysis

Cells were lysed with protease inhibitor and phosphatase inhibitor (Sigma–Aldrich) in lysis buffer (CST), and then the cell lysates were incubated with antibodies at 4 °C for 2 h. Next, protein G agarose beads (Santa Cruz, CA, USA) were added, and the mixture was incubated at 4 °C overnight with gentle shaking. Then, the beads were washed four times with lysis buffer and incubated in loading buffer at 100 °C for 10 min, and Western blot assays were then performed. Primary antibodies against PCDH1 (1:1000, #SAB2108197, Sigma–Aldrich), p65 (1:1000, #8242, CST), histone H3 (1:2000, #4499, CST), phospho-IKKα/β (1:1000, #2697, CST), IKKβ (1:1000, #2678, CST), phospho-IκBα (1:1000, #2859, CST), IκBα (1:1000, #4814, CST), Flag tag (1:2000, #F3165, Sigma–Aldrich), Myc tag (1:2000, #2276, CST) and GAPDH (1:2000, sc-47724, Santa) were used. Finally, an enhanced chemiluminescence kit (Amersham Biosciences, Piscataway, NJ, USA) was used according to the manufacturer’s instructions.

### Immunofluorescence

PDAC cells were seeded into 6-well plates at a density of 5 × 10^4^ cells per well. The cells were fixed, permeabilized, blocked, and incubated with anti-p65 antibody (1:200, #8242, CST) overnight at 4 °C. The cells were washed three times with PBS buffer and incubated with Alexa Fluor 488-conjugated secondary antibody (1:200, #4412, CST) in 3% bovine serum albumin (BSA) for 1 h at room temperature. Finally, the nucleus was stained with DAPI (Sigma-Aldrich) after washing with PBS three times. The images were visualized under a fluorescence microscope (Carl Zeiss AG).

### Microarray analyses

RNA was extracted from tumour cells as described above. RNA quality was measured with an Agilent 2100 Bioanalyzer (Agilent Technologies) and RNase free agarose gel electrophoresis. Then, RNA was amplified and labelled with a Low Input Quick Amp WT Labelling Kit (Cat# 5190-2943, Agilent Technologies). The data were extracted with Feature Extraction software 10.7 (Agilent Technologies). The raw data were normalized with a Quantile algorithm in the GeneSpring Software 11.0 (Agilent Technologies). The microarray data have been deposited in the Gene Expression Omnibus repository under accession codes GSE201971. For GSEA, normalized expression data were analyzed and visualized with GSEA software (http://www.broadinstitute.org/gsea/).

### Cell viability assays

For colony formation assays, PCDH1-silenced cells and control cells were seeded into 6-well plates at a density of 1000 cells per well and cultured for 14 days. Five hundred cells per well were used when performing cells overexpressing PCDH1. Subsequently, the cells were fixed and stained with 0.5% crystal violet in methanol, and colonies consisting of more than 50 cells were counted. Cell growth curves were plotted on the basis of the cell viability values obtained through the MTT method.

### Transwell migration and invasion assays

For PCDH1-downregulated groups and corresponding control cells, 5 × 10^5^ cells in serum-free medium were seeded into Boyden chambers (8-μm pores, BD Falcon, San Jose, CA, USA) with and without Matrigel (#356234, BD Falcon) for migration and invasion assays, respectively. Then, the chambers were placed in 24-well plates with medium containing 10% foetal bovine serum (FBS). After 20 h, cells on the underside of the filter membrane were fixed and stained with 0.5% crystal violet in methanol. The cells were counted under a microscope. For PCDH1-overexpressing groups, 2 × 10^5^ cells were used for these assays.

### Side population assay

SP assays were conducted as previously reported [47–49]. A density of 1 × 10^6^ cells/ml was suspended in medium with Hoechst 33342 (Life Technologies) at a final concentration of 5 μg/ml, and the verapamil control groups were simultaneously treated with 100 μM verapamil. Then, the samples were incubated in a 37 °C water bath for 90 min with gentle mixing every 5 min. After incubation, the cells were resuspended in PBS with 1 μg/ml propidium iodide (PI; Life Technologies) and filtered through a 40-μm cell strainer (BD Falcon). Finally, flow cytometry analysis was performed to assess the proportion of cells exhibiting the SP phenotype.

### Dual-luciferase reporter assay

Cells were co-transfected with the indicated plasmids, luciferase reporters and pRL-TK (Promega, Madison, WI, USA). After 24 h, luciferase activity was detected according to the assay kit manufacturer’s instructions (Promega, Madison, WI, USA).

### Animal experiments

Animal experiments were approved by the Animal Ethics Committee of Zhongshan People’s Hospital. Female BALB/c nude mice (4 weeks old and 15–18 g) were purchased from Cyagen (Suzhou, China). Mice were allocated randomly to each treatment group using random number table (*n* = 10 each group). Different groups were processed identically, and animals in different groups were exposed to the same environment. The feeding of mice was carried out by the animal laboratory specialist, and the researchers remained blind until the end of the experiments. To establish the xenograft model, 1 × 106 cells were injected subcutaneously in the mice. The mice were sacrificed 4 weeks later, and the tumours were dissected. The following formula was used to evaluate the tumour volume: *V* = (length × width2)/2. For the hepatic metastasis model, the mice were anaesthetized with isoflurane, and the spleen was pulled out of the abdominal cavity by laparotomy. A total of 1 × 10^6^ cells were inoculated into the spleen. In the lung metastasis model, mice were injected with 1 × 10^6^ cells through the tail vein. To establish the inguinal lymph node metastasis model, 1 × 10^6^ cells were injected into the footpads of mice. After 6 weeks, the mice were sacrificed, and the liver/lungs/inguinal lymph nodes were removed for HE staining and microscopy examination, as previously described [50, 51].

### Statistical analysis

SPSS software version 21.0 (SPSS Inc, Chicago, IL, USA) was used for the statistical analysis. *χ*^2^-tests were performed to evaluate the correlation between PCDH1 expression and clinicopathological parameters. A Kaplan–Meier analysis was performed to determine patient survival. A Cox regression model was used for the multivariate survival analysis. Pearson’s test was performed for the correlation analysis. Between-group variations in this study were evaluated by the use of the Student’s 2-tailed *t* test. The variance between the groups that are being statistically compared is similar. *P* < 0.05 was considered to be statistically significant.

### Supplementary information

Supplementary Figure

Figure S1

Figure S2

Figure S3

Figure S4

Figure S5

Figure S6

Figure S7

Figure S8

Supplementary Methods and Materials

aj-checklist

Original Data File

Table S1

Table S2

Table S3

## Data Availability

The datasets used and/or analysed in the current study are available from the corresponding author on reasonable request.
