# Correction: PCDH1 promotes progression of pancreatic ductal adenocarcinoma via activation of NF-κB signalling by interacting with KPNB1

**DOI:** 10.1038/s41419-023-06403-w

**Published:** 2024-01-09

**Authors:** Zhihua Ye, Yingyu Yang, Ying Wei, Lamei Li, Xinyi Wang, Junkai Zhang

**Affiliations:** Department of Medical Oncology Center, Zhongshan City People’s Hospital, 528403 Zhongshan City, Guangdong Province PR China

**Keywords:** Oncogenes, Prognostic markers

Correction to: *Cell Death and Disease* 10.1038/s41419-022-05087-y, published online 21 July 2022

In this article, during the process of reorganizing the data in the article, the author found an error where the two adjacent images in Figure 2D were duplicated. The mistake occurred in the process of image organization. They have replaced the correct image.
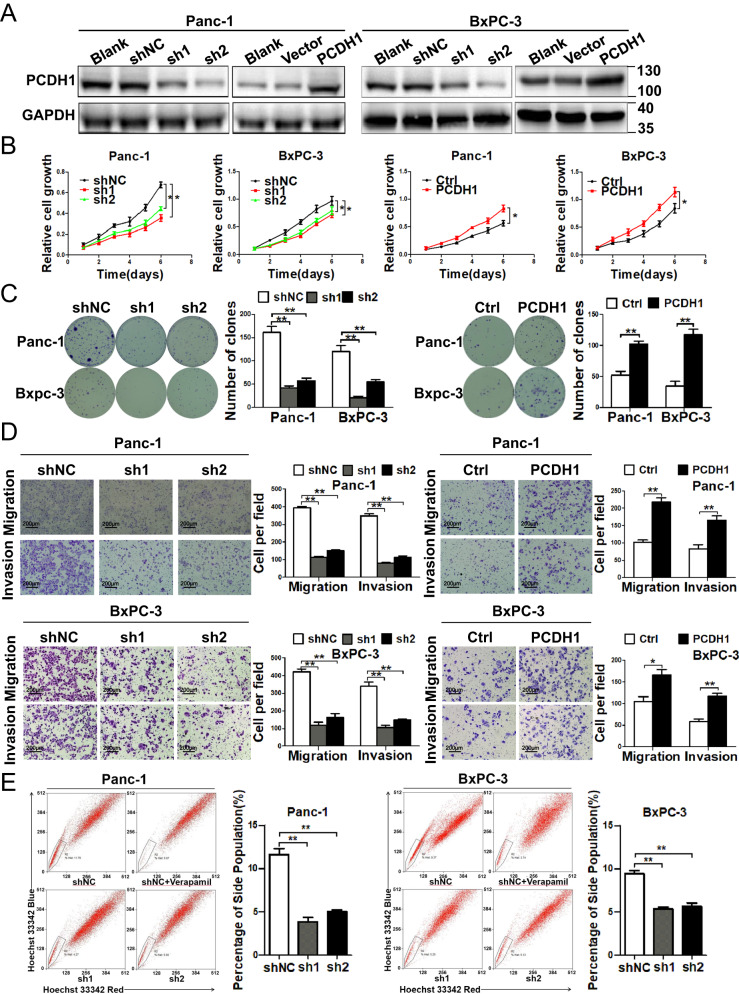


It should be read:
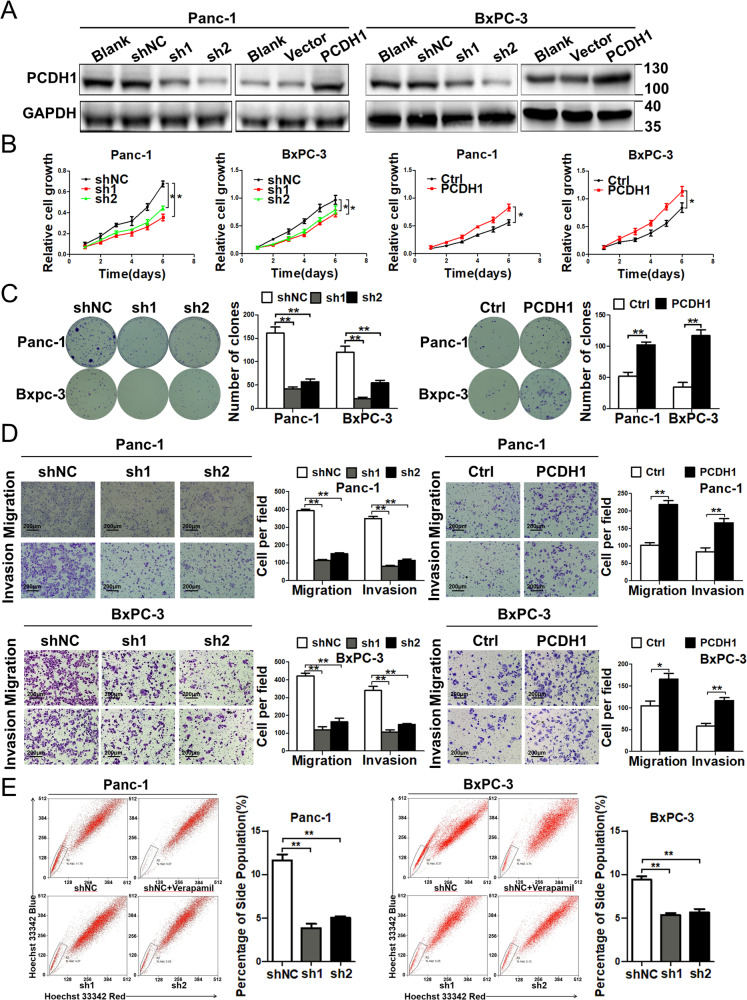


The original article has been corrected.

