# Supplementary material for: PCDH1 promotes progression of pancreatic ductal adenocarcinoma via activation of NF-κB signalling by interacting with KPNB1
Source: Cell Death Dis. 2022 Jul 21;13(7):633. doi: 10.1038/s41419-022-05087-y (PMC9304345; doi:10.1038/s41419-022-05087-y)
Supplement: Supplementary file 1 — Supplementary Figure [file 41419_2022_5087_MOESM1_ESM.docx]

**Supplementary figure**


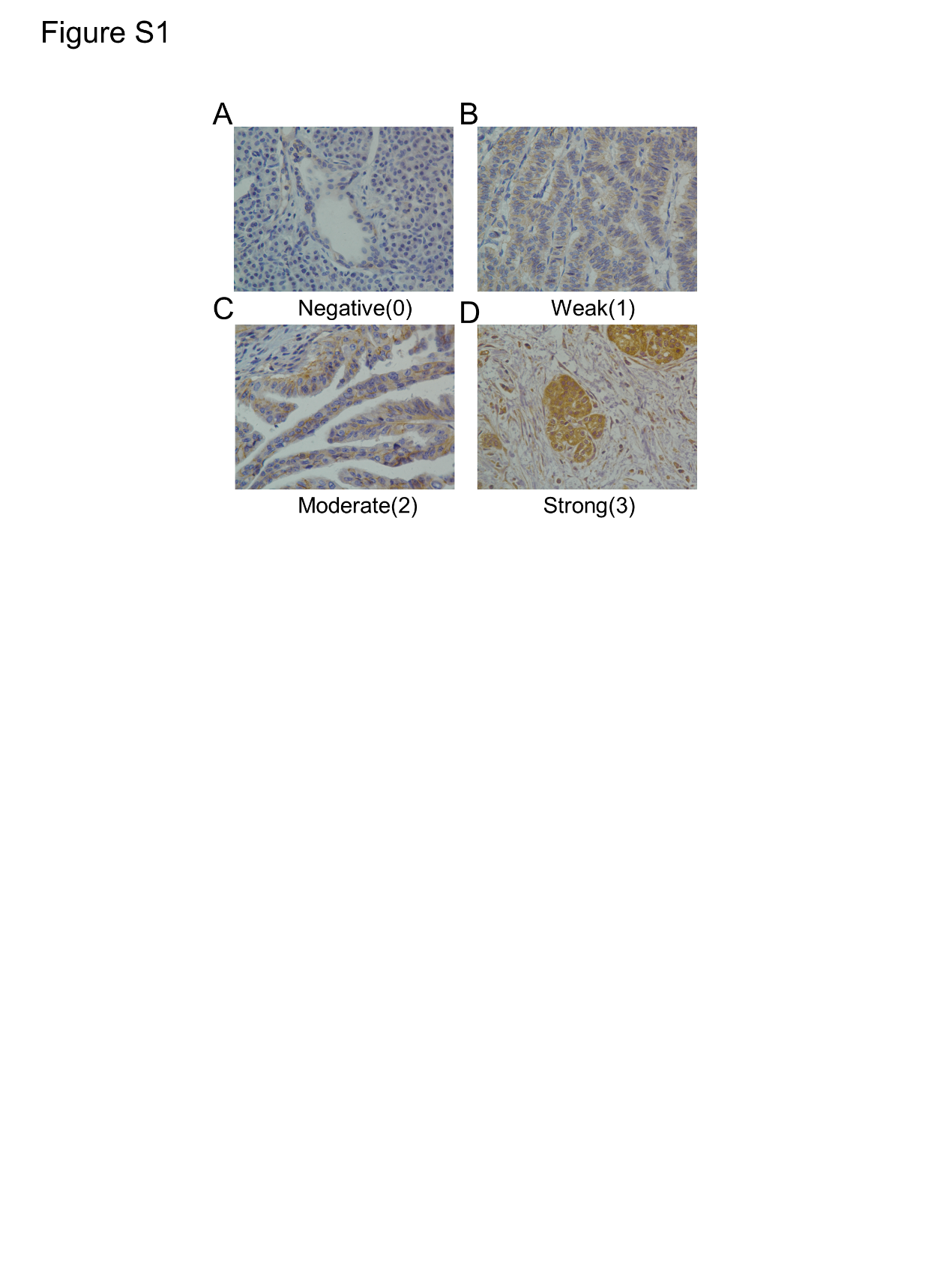


**Figure S1.** Representative images(200×magnification) of IHC staining for PCDH1 in PDAC tissues. PCDH1 expression was scored from 0 to 3. A score of 0 represents negative staining(A), a score of 1 indicates weak positive staining(B), a score of 2 indicates moderate positive staining(C), and a score of 3 represents strong positive staining(D).


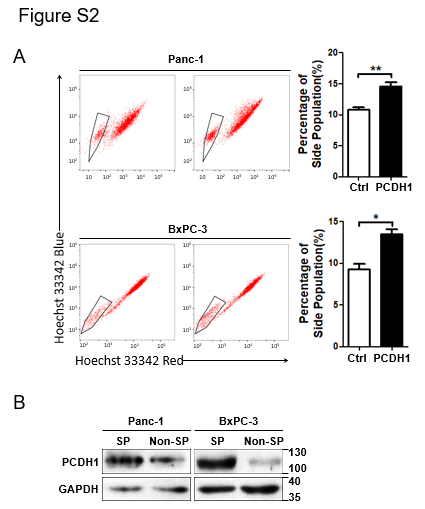


**Figure S2.** (A)Representative images showing the effects of PCDH1 overexpression on SP phenotype acquisition. The results are presented as the mean ± SD of three independent experiments. The data were assessed by Student’s 2-tailed t test. **P* < 0. 05, ***P* < 0. 01. (B)Evaluation of PCDH1 expression in SP and non-SP cells in PDAC cell lines separated by flow cytometry analysis.


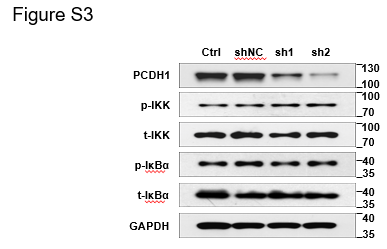


**Figure S3.** Western blot analysis of the phosphorylation levels of IKK or IκBα in Panc-1 silencing PCDH1. Three independent experiments were conducted.


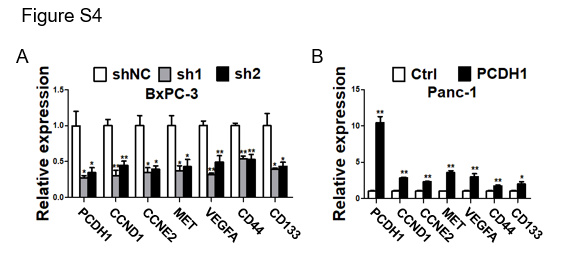


**Figure S4.** qRT-PCR analysis of genes related to growth, metastasis and CSCs in PDAC cells after PCDH1 down or up regulation. The results presented are the mean ± SD of three independent experiments. The data were assessed with student’s 2-tailed t test. *P＜0. 05, **P＜0. 01.


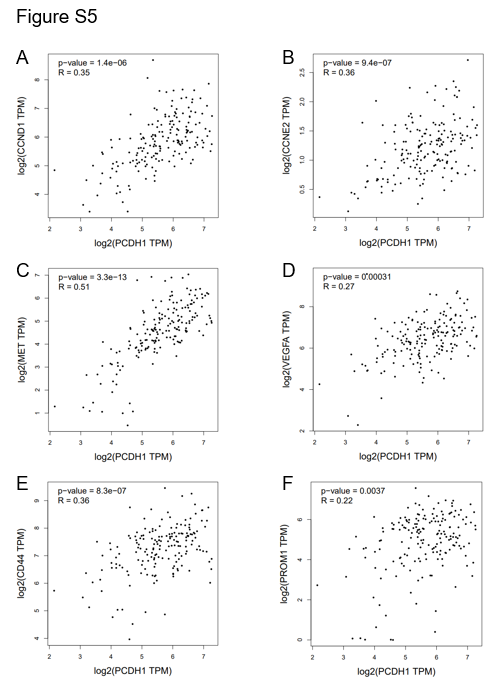


**Figure S5.** The correlation between the mRNA levels of CCND1(A), CCNE2(B), MET(C), VEGFA(D), CD44(E), CD133(F) and PCDH1 in TCGA dataset (GEPIA online program).


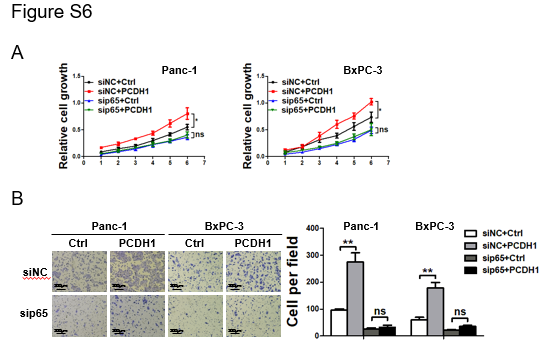


**Figure S6.** PCDH1 promotes proliferation and migration of PDAC cells via NF-κB pathway. MTT (A) and Transwell assays (B) were performed to detect the effects of PCDH1 expression on PDAC cell proliferation and migration after p65 silencing. The results presented are the mean ± SD of three independent experiments. The data were assessed by Student’s 2-tailed t test. **P* < 0. 05, ***P* < 0. 01.


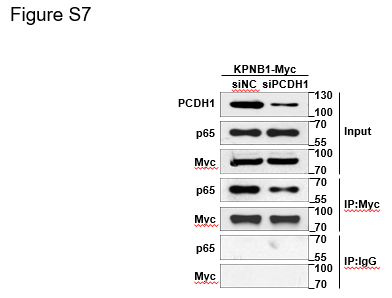


**Figure S7.** The binding ability of p65 and KPNB1 after silencing PCDH1 was detected by co-IP western blot. Three independent experiments were conducted.


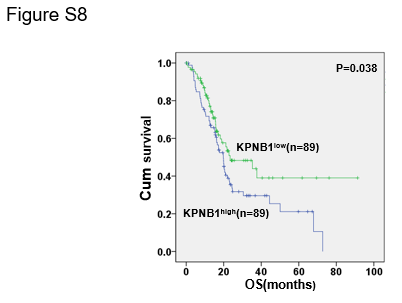


**Figure S8.** High expression of *PCDH1* in the TCGA dataset was correlated with worse prognosis in PDAC (GEPIA online program).
