# Supplementary figures and images for: PCDH1 promotes progression of pancreatic ductal adenocarcinoma via activation of NF-κB signalling by interacting with KPNB1
Source: Cell Death Dis. 2022 Jul 21;13(7):633. doi: 10.1038/s41419-022-05087-y (PMC9304345; doi:10.1038/s41419-022-05087-y)

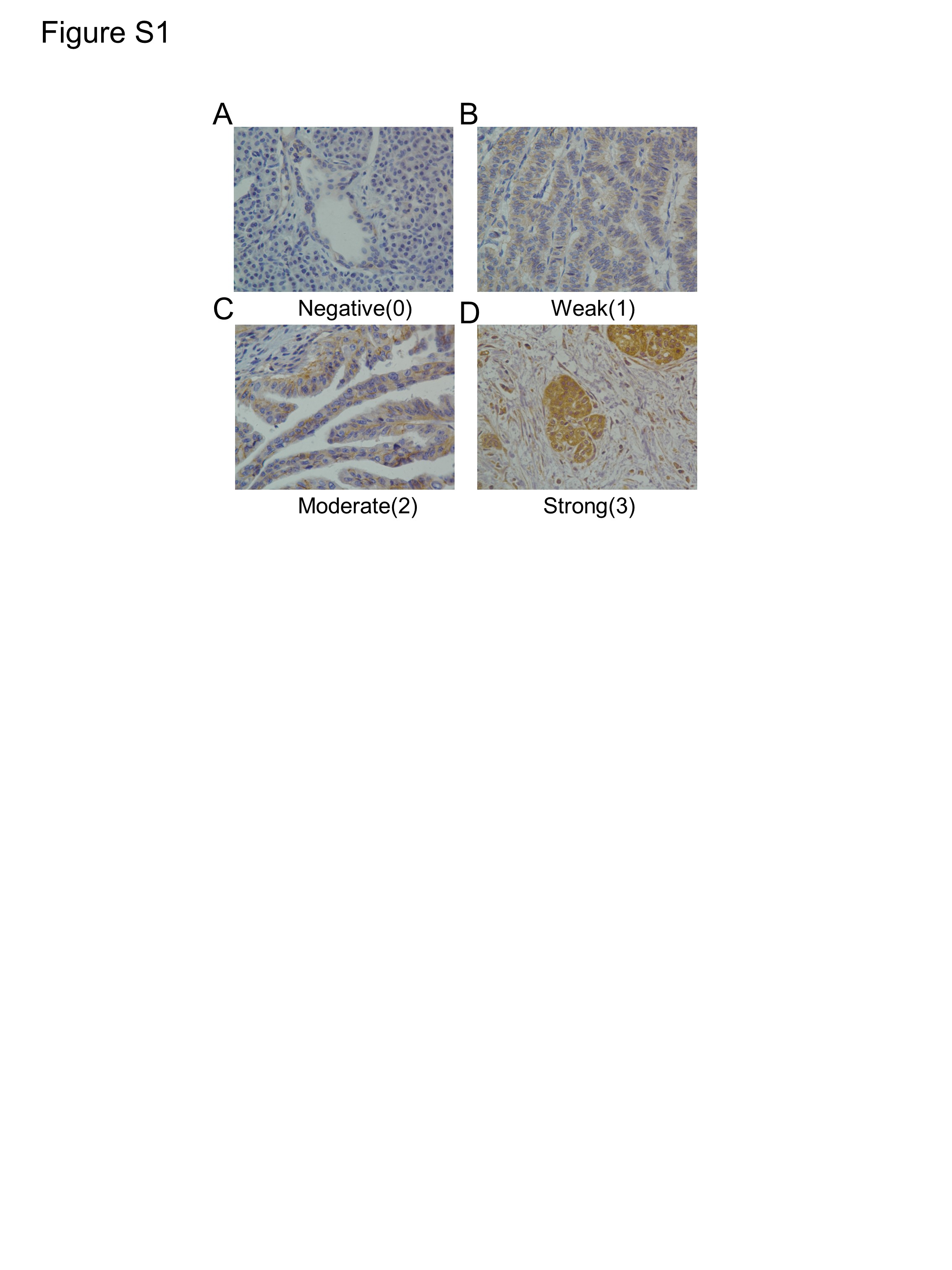

Supplement: Supplementary file 2 — Figure S1 [file 41419_2022_5087_MOESM2_ESM.jpg]

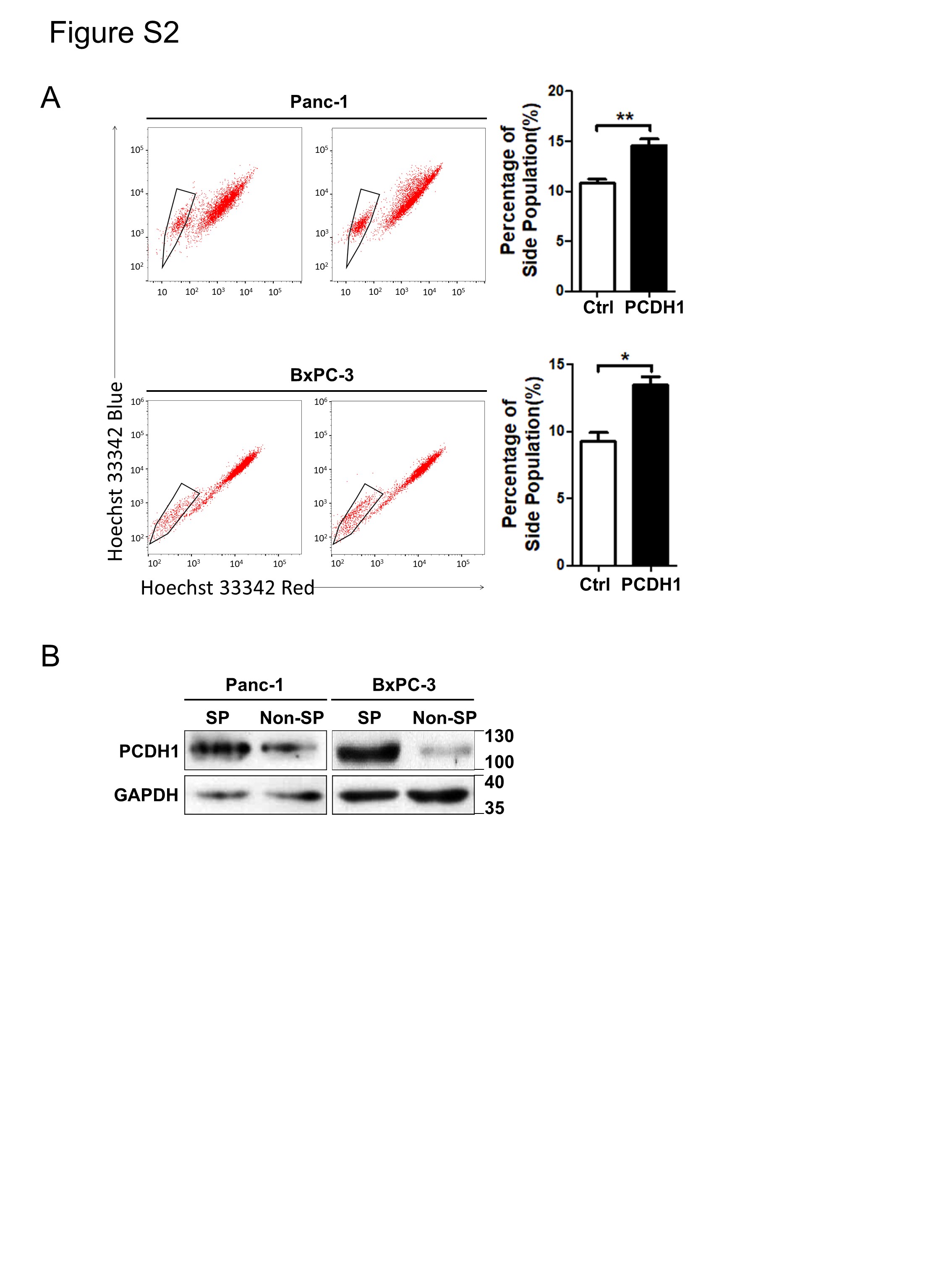

Supplement: Supplementary file 3 — Figure S2 [file 41419_2022_5087_MOESM3_ESM.jpg]

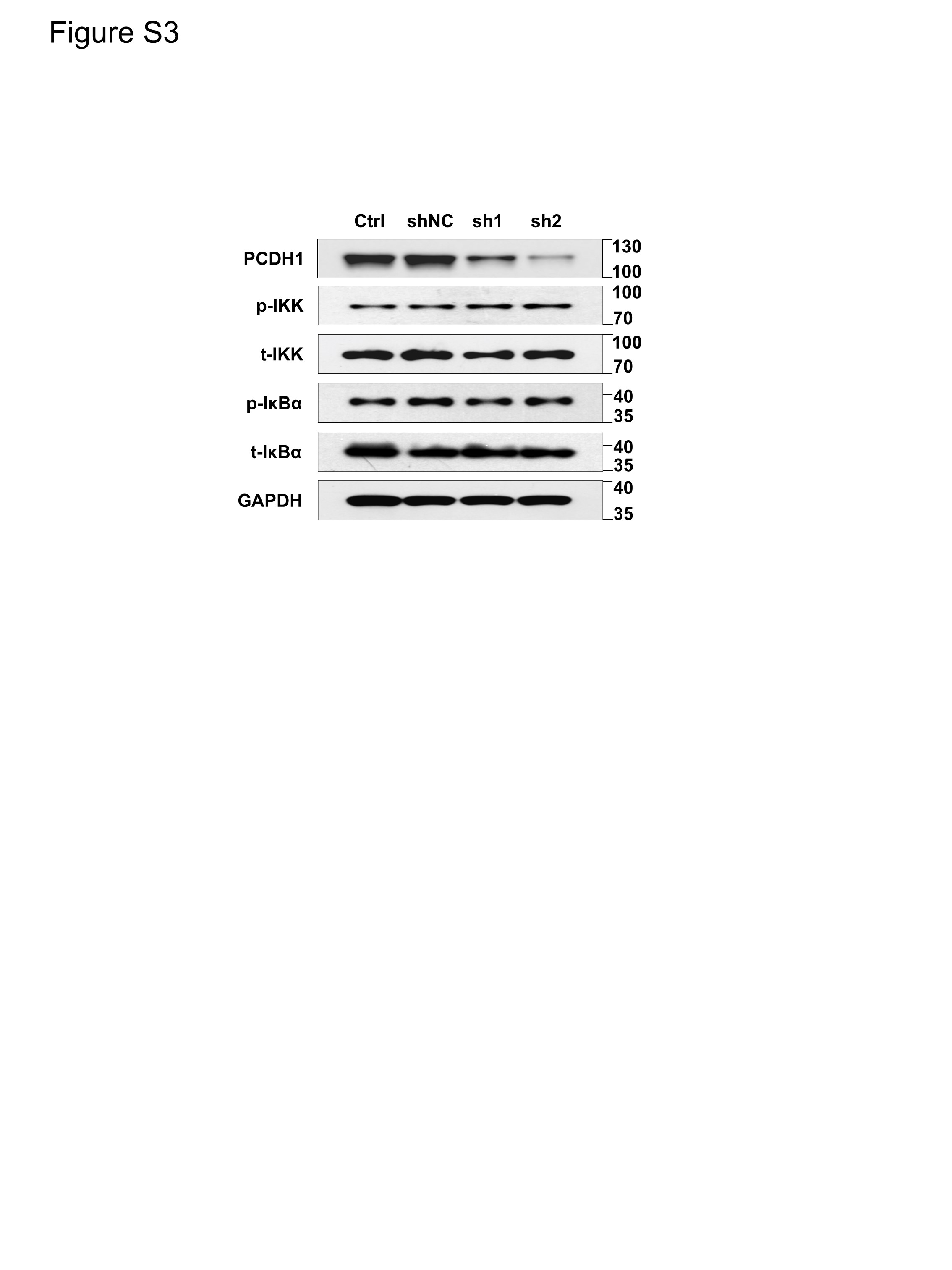

Supplement: Supplementary file 4 — Figure S3 [file 41419_2022_5087_MOESM4_ESM.jpg]

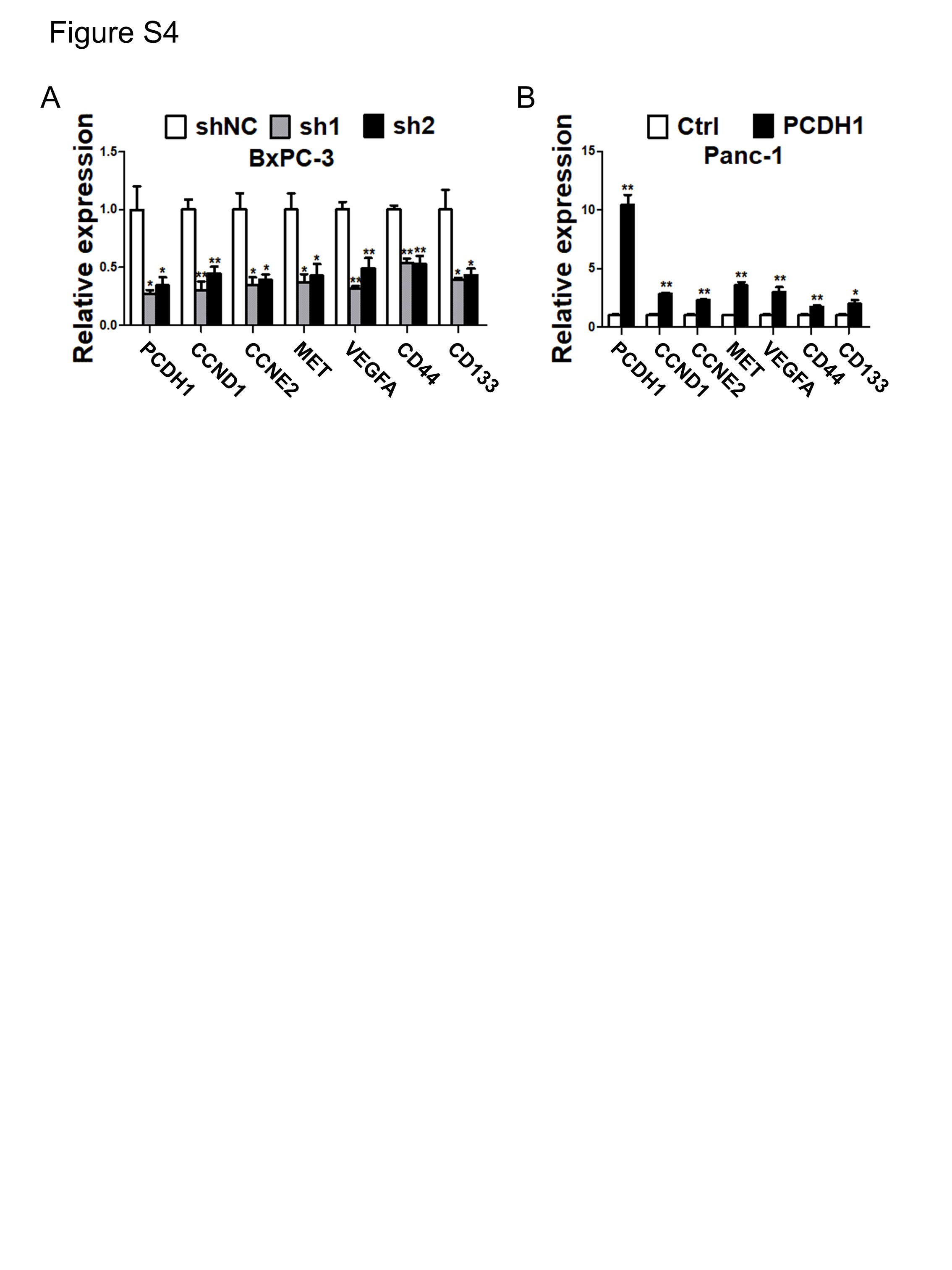

Supplement: Supplementary file 5 — Figure S4 [file 41419_2022_5087_MOESM5_ESM.jpg]

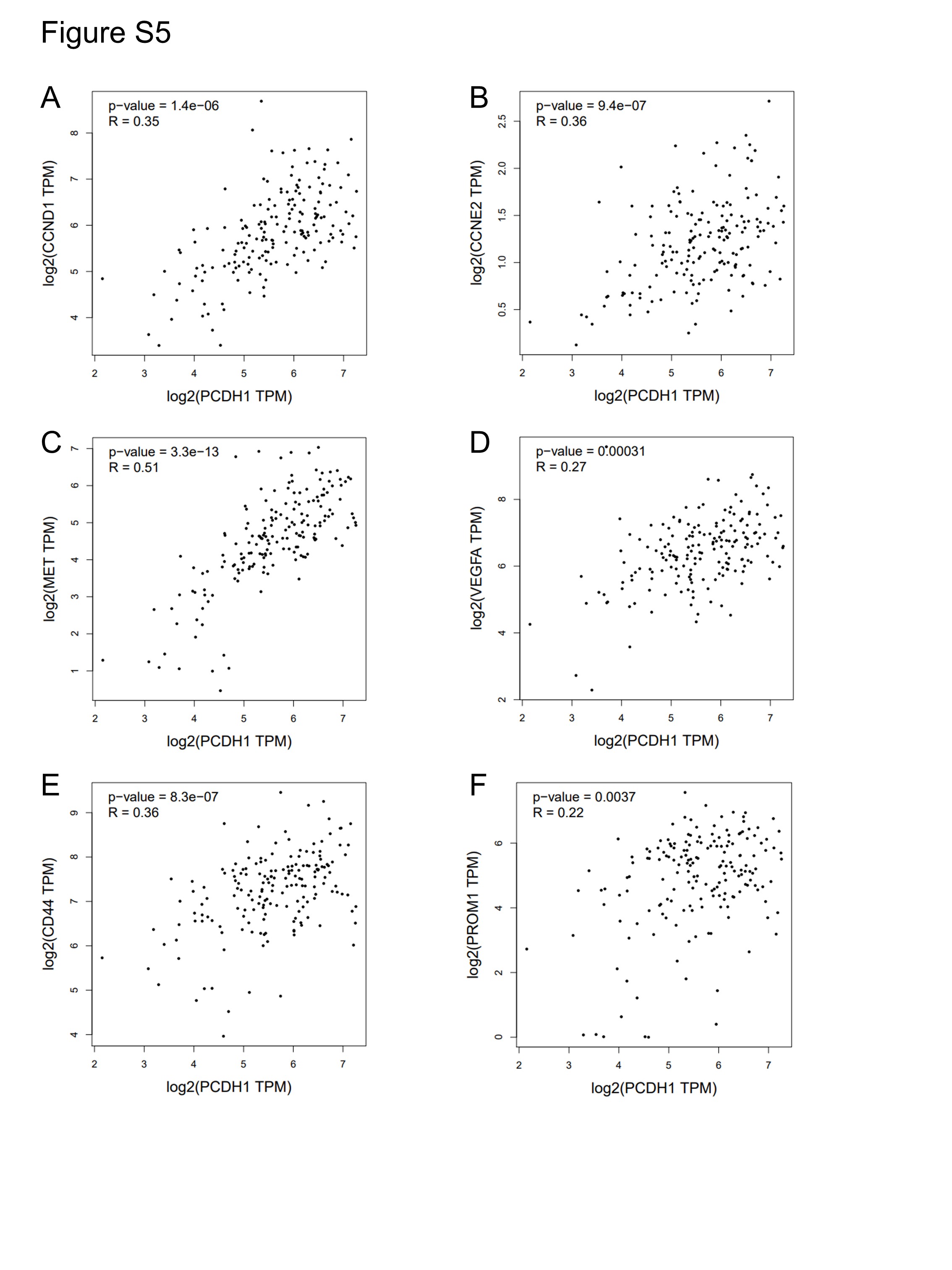

Supplement: Supplementary file 6 — Figure S5 [file 41419_2022_5087_MOESM6_ESM.jpg]

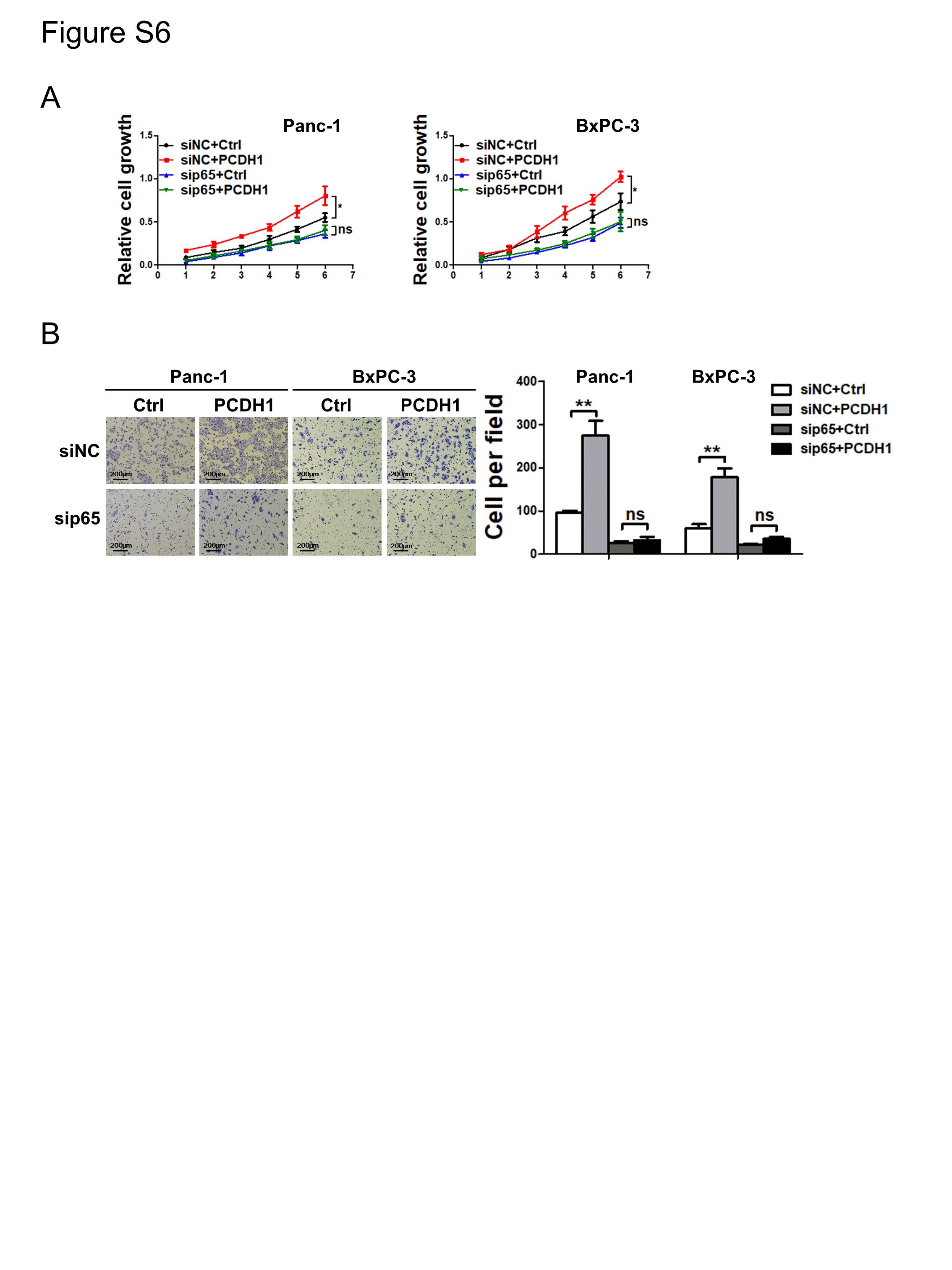

Supplement: Supplementary file 7 — Figure S6 [file 41419_2022_5087_MOESM7_ESM.jpg]

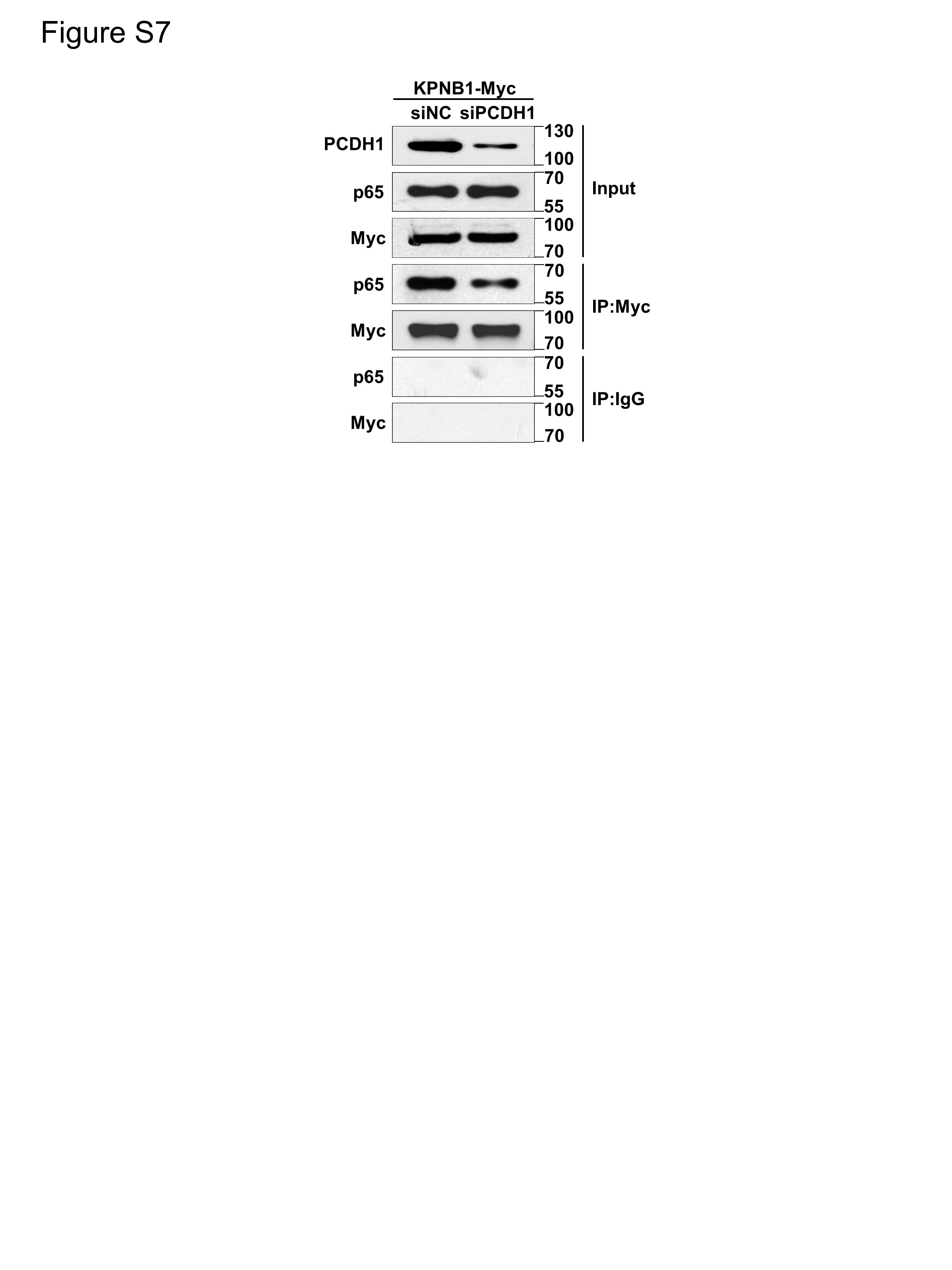

Supplement: Supplementary file 8 — Figure S7 [file 41419_2022_5087_MOESM8_ESM.jpg]

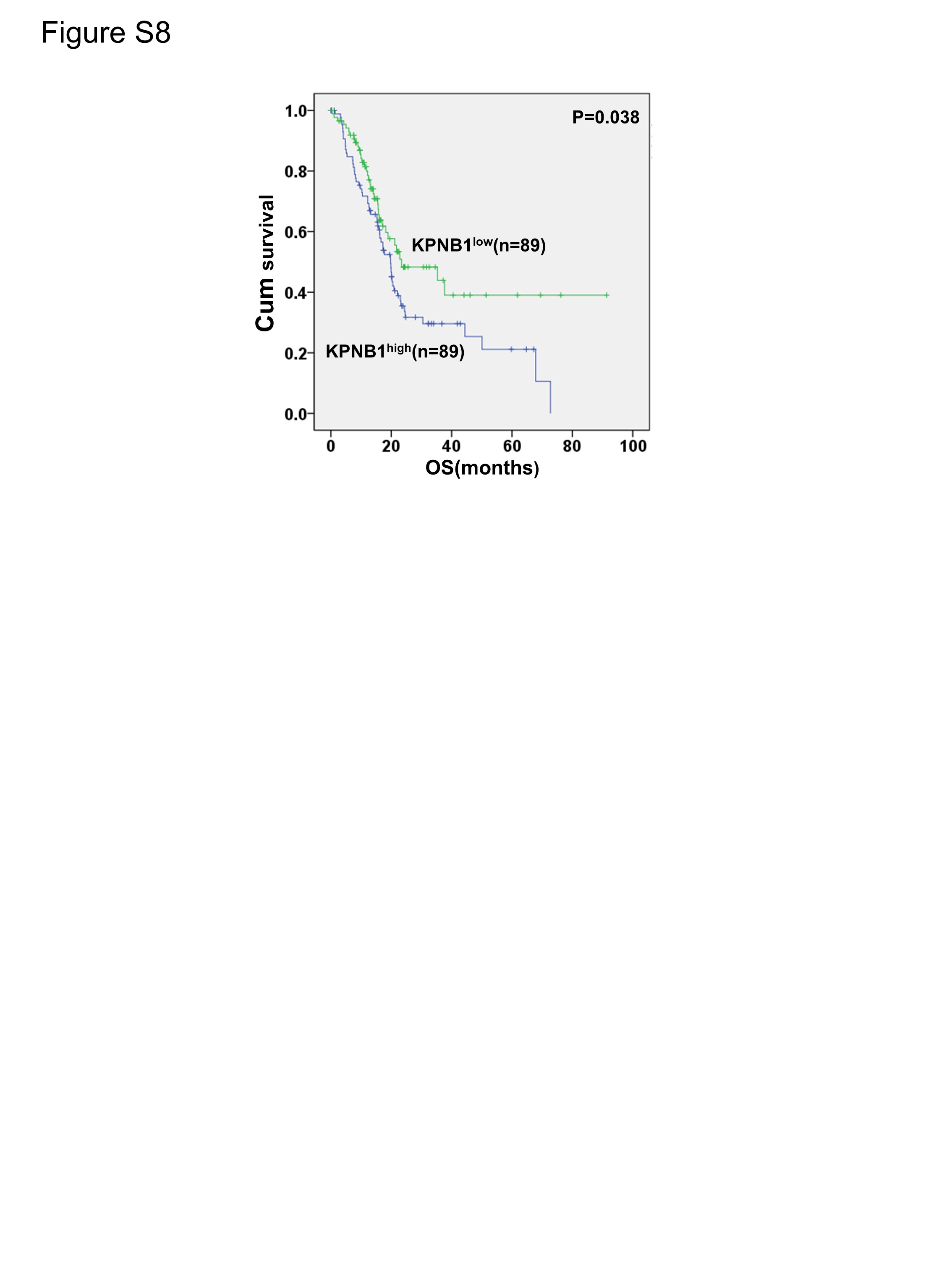

Supplement: Supplementary file 9 — Figure S8 [file 41419_2022_5087_MOESM9_ESM.jpg]
