# Supplementary material for: PCDH1 promotes progression of pancreatic ductal adenocarcinoma via activation of NF-κB signalling by interacting with KPNB1
Source: Cell Death Dis. 2022 Jul 21;13(7):633. doi: 10.1038/s41419-022-05087-y (PMC9304345; doi:10.1038/s41419-022-05087-y)
Supplement: Supplementary file 10 — Supplementary Methods and Materials [file 41419_2022_5087_MOESM10_ESM.docx]

**Patient tissue specimens and clinicopathological characteristics**

Sample size was determined on the basis of similar research reported in the literature. Ninety-seven PDAC tissues were collected at Zhongshan People's Hospital (Zhongshan, China). Another 100 specimens, including PDAC tissues and their adjacent normal tissues, were in tissue in microarrays (TMAs) purchased from Shanghai Outdo Biotech. All patients did not accept neoadjuvant therapy before operation, and there were detailed follow-up data. The patients were followed-up once every 3 months during the first 2 years, once every 6 months during the third and fourth year, and once a year from the fifth year postoperatively. The patients who did not have follow-up information were excluded from this study. The collected tissue specimens conformed to the criteria that they contained matched tumors (percentage of tumor cells > 70%) and corresponding normal mucosal tissue (>5 cm laterally from the edge of the tumor region). The use of clinical specimens for research purposes was approved by the Ethics Committee of Zhongshan People's Hospital, and the patients were informed prior to the use of clinical material.
