# Supplementary material for: PCDH1 promotes progression of pancreatic ductal adenocarcinoma via activation of NF-κB signalling by interacting with KPNB1
Source: Cell Death Dis. 2022 Jul 21;13(7):633. doi: 10.1038/s41419-022-05087-y (PMC9304345; doi:10.1038/s41419-022-05087-y)

**Original data of western blot**

Figure 1C


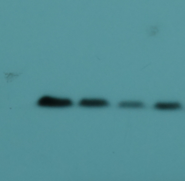


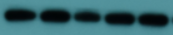


Figure 2A


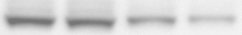

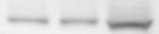


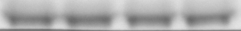

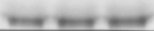


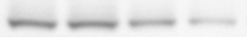

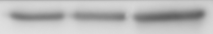


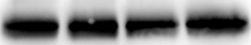

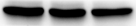


Figure 4E


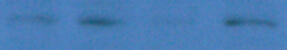

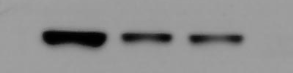

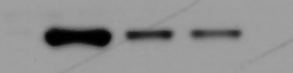


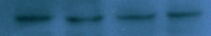

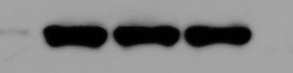

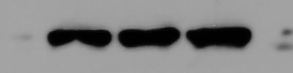


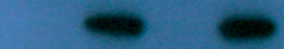

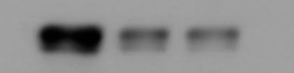

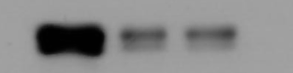


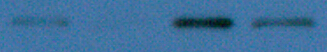

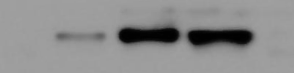

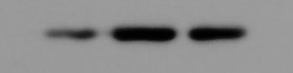


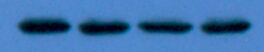

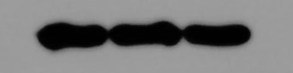

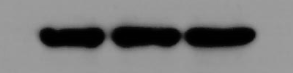


Figure 6A Figure 6B


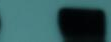

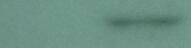

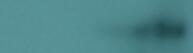


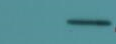

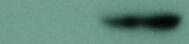

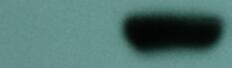


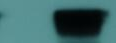

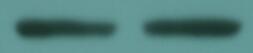

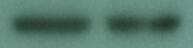


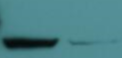

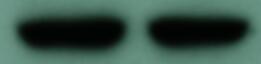

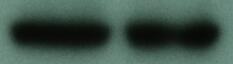


Figure 6C


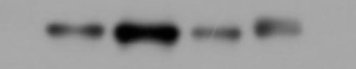

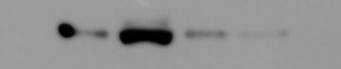


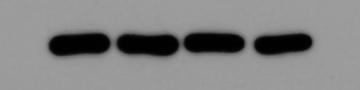

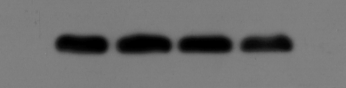


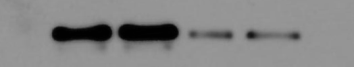

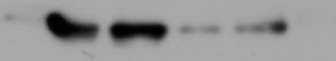


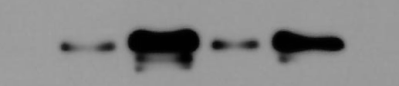

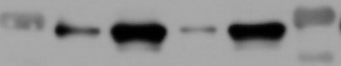


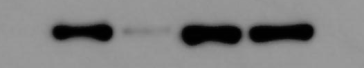

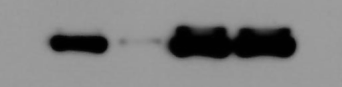


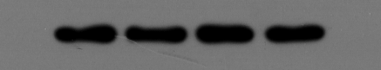

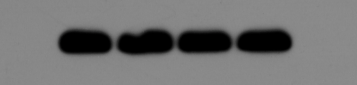


Figure S3


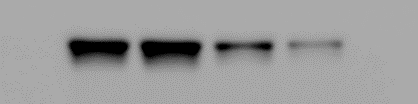


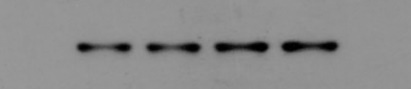


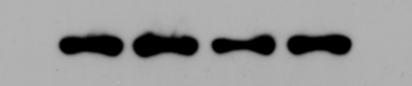


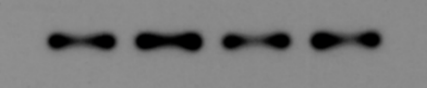


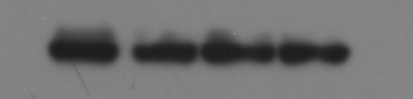


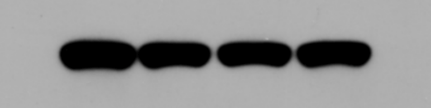


Figure S7


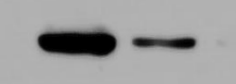


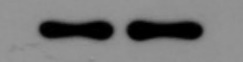


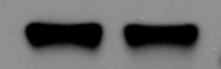


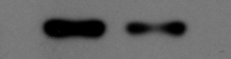


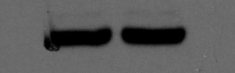


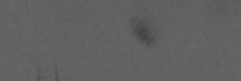


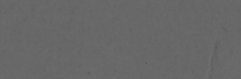


**Original data of flow cytometry(Figure 2E)**

Panc-1

**
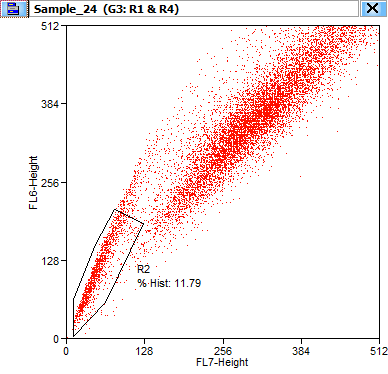

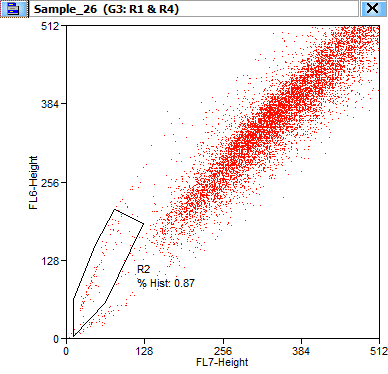
**

**
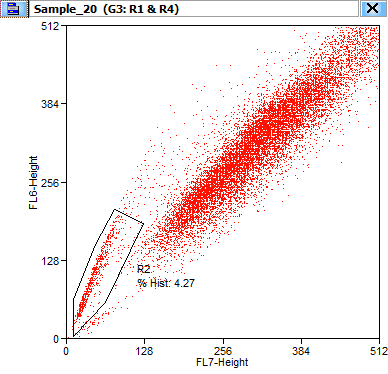

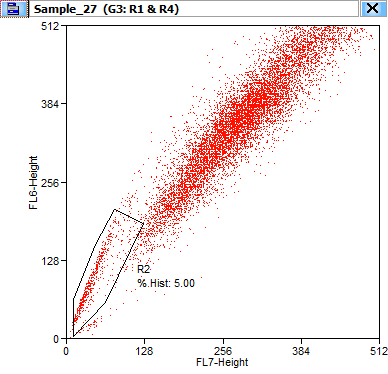
**

BxPC-3


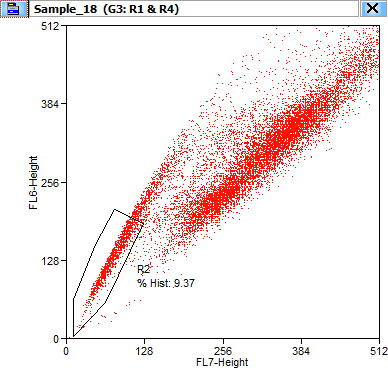

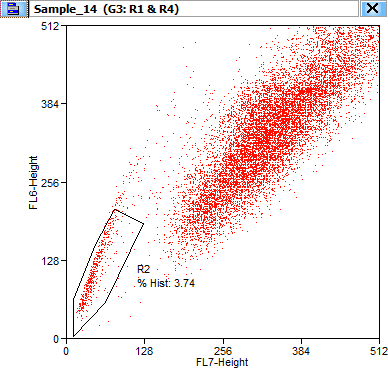


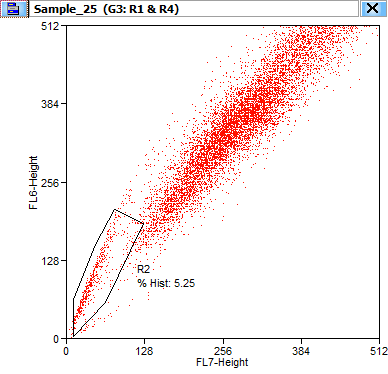

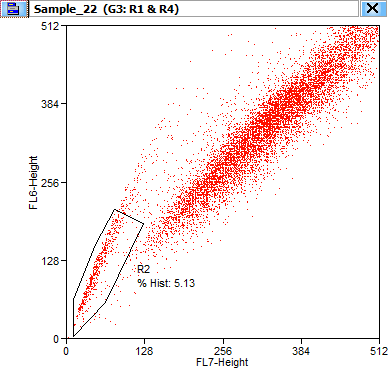

Supplement: Supplementary file 12 — Original Data File [file 41419_2022_5087_MOESM12_ESM.docx]
