# Supplementary material for: PCDH1 promotes progression of pancreatic ductal adenocarcinoma via activation of NF-κB signalling by interacting with KPNB1
Source: Cell Death Dis. 2022 Jul 21;13(7):633. doi: 10.1038/s41419-022-05087-y (PMC9304345; doi:10.1038/s41419-022-05087-y)
Supplement: Supplementary file 13 — Table S1 [file 41419_2022_5087_MOESM13_ESM.docx]

**Table S1.** Candidate proteins interact with PCDH1.

| **Gene names** | **Score** |
| --- | --- |
| PCDH1 | 238.03 |
| TUBA1C; TUBA1A; TUBA1B; TUBA2 | 170.94 |
| FAT1 | 120.92 |
| KPNB1 | 110.77 |
| RPL7A; RP-L7a | 88.584 |
| HSPA8; HEL-S-72p; HSPA2 | 80.814 |
| PS1TP5BP1; ACTG1; ACTB | 75.24 |
| EEF1A1; EEF1A1P5; EEF1A1L14; EEF1A2 | 72.42 |
| HNRNPM; ORF | 64.097 |
| SYNCRIP | 59.5 |
| MATR3; DKFZp686K23100 | 58.392 |
| ATAD3A; ATAD3B; ATAD3C | 52.749 |
| SLC25A6 | 48.531 |
| KEAP1 | 44.042 |
| hCG_1994130; RPS15A | 40.273 |
