# Supplementary material for: PCDH1 promotes progression of pancreatic ductal adenocarcinoma via activation of NF-κB signalling by interacting with KPNB1
Source: Cell Death Dis. 2022 Jul 21;13(7):633. doi: 10.1038/s41419-022-05087-y (PMC9304345; doi:10.1038/s41419-022-05087-y)
Supplement: Supplementary file 14 — Table S2 [file 41419_2022_5087_MOESM14_ESM.docx]

**Table S2.** siRNA and short hairpin RNA target sequences.

| **Name** | Sequences |
| --- | --- |
| PCDH1 siRNA1 and shRNA1 | 5'-GCTGAGCTGATCTACAGCATT-3' |
| PCDH1 siRNA2 and shRNA2 | 5'-GCTCTAATGCTGAGCTGGTTT-3' |
| KPNB1 shRNA | 5'-GCGCTGTTAGACATGAGCTAA-3' |
| p65 siRNA | 5'-CGGATTGAGGAGAAACGTAAA-3' |
