# Supplementary material for: PCDH1 promotes progression of pancreatic ductal adenocarcinoma via activation of NF-κB signalling by interacting with KPNB1
Source: Cell Death Dis. 2022 Jul 21;13(7):633. doi: 10.1038/s41419-022-05087-y (PMC9304345; doi:10.1038/s41419-022-05087-y)
Supplement: Supplementary file 15 — Table S3 [file 41419_2022_5087_MOESM15_ESM.docx]

**Table S3.** The primers for qPCR.

| **Name** | **Forward** | **Reverse** |
| --- | --- | --- |
| IL-6 | AAGCCAGAGCTGTGCAGATGAGTA | TGTCCTGCAGCCACTGGTTC |
| IL-8 | ACACTGCGCCAACACAGAAATTA | TTTGCTTGAAGTTTCACTGGCATC |
| TNFA | CAGGGGCCACCACGCTCTTC | CTTGGGGCAGGGGCTCTTGAC |
| PCDH1 | GGAACAGAGGGTTGGATCAGT | GGCTCGAAATCGAACAGTAGAA |
| CCND1 | AGTTGTTGGGGCTCCTCAG | AGACCTTCGTTGCCCTCTGT |
| CCNE2 | TCAAGACGAAGTAGCCGTTTAC | TGACATCCTGGGTAGTTTTCCTC |
| VEGFA | AGGGCAGAATCATCACGAAGT | AGGGTCTCGATTGGATGGCA |
| MET | GGCTCCTGGCAAAAGGTCA | CTGCGTAGTTGTGCTGATGT |
| CD44 | CTGCCGCTTTGCAGGTGTA | CATTGTGGGCAAGGTGCTATT |
| CD133 | AGTCGGAAACTGGCAGATAGC | GGTAGTGTTGTACTGGGCCAAT |
| GAPDH | AGAAGGCTGGGGCTCATTTG | AGGGGCCATCCACAGTCTTC |
